# Effectiveness of a Vaping Cessation Text Message Program Among Young Adult e-Cigarette Users: A Randomized Clinical Trial

**DOI:** 10.1001/jamainternmed.2021.1793

**Published:** 2021-05-17

**Authors:** Amanda L. Graham, Michael S. Amato, Sarah Cha, Megan A. Jacobs, Mia M. Bottcher, George D. Papandonatos

**Affiliations:** 1 Innovations Center, Truth Initiative, Washington, DC; 2 Department of Medicine, Mayo Clinic College of Medicine and Science, Rochester, Minnesota; 3 Department of Oncology, Georgetown University Medical Center/Cancer Prevention and Control Program, Lombardi Comprehensive Cancer Center, Washington, DC; 4 Center for Statistical Sciences, School of Public Health, Brown University, Providence, Rhode Island

## Abstract

**Importance:**

e-Cigarettes are the most commonly used tobacco product among young adults (YAs). Despite the harms of nicotine exposure among YAs, there are few, if any, empirically tested vaping cessation interventions available.

**Objective:**

To determine the effectiveness of a text message program for vaping cessation among YAs vs assessment-only control.

**Design, Setting, and Participants:**

A parallel, 2-group, double-blind, individually randomized clinical trial was conducted from December 2019 to November 2020 among YA e-cigarette users. Eligible individuals were US residents aged 18 to 24 years who owned a mobile phone with an active text message plan, reported past 30-day e-cigarette use, and were interested in quitting in the next 30 days. Participants were recruited via social media ads, the intervention was delivered via text message, and assessments were completed via website or mobile phone. Follow-up was conducted at 1 and 7 months postrandomization; follow-up data collection began January 2020 and ended in November 2020. The study was prespecified in the trial protocol.

**Interventions:**

All participants received monthly assessments via text message about e-cigarette use. The assessment-only control arm (n = 1284) received no additional intervention. The active intervention arm (n = 1304) also received This is Quitting, a fully automated text message program for vaping cessation that delivers social support and cognitive and behavioral coping skills training.

**Main Outcomes and Measures:**

The primary outcome was self-reported 30-day point prevalence abstinence (ppa) at 7 months analyzed under intention-to-treat analysis, which counted nonresponders as vaping. Secondary outcomes were 7-day ppa under intention-to-treat analysis and retention weighted complete case analysis of 30-day and 7-day ppa.

**Results:**

Of the 2588 YA e-cigarette users included in the trial, the mean (SD) age was 20.4 (1.7) years, 1253 (48.4%) were male, 2159 (83.4%) were White, 275 (10.6%) were Hispanic, and 493 (19.0%) were a sexual minority. Most participants (n = 2129; 82.3%) vaped within 30 minutes of waking. The 7-month follow-up rate was 76.0% (n = 1967), with no differential attrition. Abstinence rates were 24.1% (95% CI, 21.8%-26.5%) among intervention participants and 18.6% (95% CI, 16.7%-20.8%) among control participants (odds ratio, 1.39; 95% CI, 1.15-1.68; *P* < .001). No baseline variables moderated the treatment-outcome relationship, including nicotine dependence.

**Conclusions and Relevance:**

Results of this randomized clinical trial demonstrated that a tailored and interactive text message intervention was effective in promoting vaping cessation among YAs. These results establish a benchmark of intervention effectiveness.

**Trial Registration:**

ClinicalTrials.gov Identifier: NCT04251273

## Key Points

**Question:**

Is a text message program for vaping cessation effective in promoting abstinence from e-cigarettes among young adults (YAs)?

**Findings:**

In this randomized clinical trial of 2588 YA e-cigarette users, at 7 months postrandomization abstinence rates were 24.1% among participants assigned to the text message intervention and 18.6% among participants assigned to an assessment-only control, which is a statistically significant difference. No baseline characteristics moderated the treatment-outcome relationship, including nicotine dependence.

**Meaning:**

A text message vaping cessation program is effective in promoting abstinence among YA e-cigarette users.

## Teaser

This randomized clinical trial determines the effectiveness of a text message program for vaping cessation among young adults aged 18 to 24 years vs assessment-only control.

## Introduction

Electronic cigarettes (e-cigarettes) are the most commonly used tobacco product among US young adults (YAs) aged 18 to 24 years.^1^ Data from the US Centers for Disease Control and Prevention show that e-cigarette use (every day or some days) among YAs increased from 5.2% in 2014 to 9.3% in 2019,^1,2^ with more than half (56%) of YA e-cigarette users reporting that they have never smoked cigarettes. The majority of e-cigarettes contain nicotine, and concentrations available in popular products have increased over the past decade.^3^ Indeed, nicotine consumption and exposure can be extremely high among YA e-cigarette users.^4,5,6^ Nicotine has negative health effects on brain development occurring into the mid-20s,^7^ including nicotine addiction, mood disorders, permanent lowering of impulse control, and deficits in attention and learning.^8^ Additionally, the aerosol produced by e-cigarettes contains known carcinogens and tiny particles that reach deep into the lungs.^9^ The effects of long-term exposure to these chemicals remain unknown. e-Cigarette use among young adults has been associated with future initiation of combustible tobacco use^10^ and with increased odds of alcohol and marijuana use.^11^

Responding to calls for research on e-cigarette cessation interventions,^12,13^ this study examined the effectiveness of a text message program for vaping cessation in a randomized clinical trial among YAs. Mobile phone ownership is ubiquitous among YAs,^14^ and text messaging is easy to use, discreet, anonymous, and a preferred communication modality in this age group.^15^ Text messaging has also been shown to be an effective intervention strategy for smoking cessation.^16,17,18^ The primary hypothesis was that participants in the active intervention arm would be more likely to be abstinent at the 7-month postrandomization primary end point than participants in an assessment-only control arm. We also examined whether any demographic, psychosocial, or substance use characteristics moderated the effectiveness of the intervention, with a particular focus on nicotine dependence as an a priori construct of interest given its well-established association with lower odds of tobacco cessation.^19^

## Methods

### Trial Design

The study was a blinded, parallel, 2-group individually randomized clinical trial that compared a tailored, interactive text message intervention with a text message–based assessment-only control among YA e-cigarette users. The study was prespecified in the trial protocol (Supplement 1).^20^ Based on short-term abstinence rates observed in the pilot trial,^20^ the study was powered to detect a treatment difference of 16% (intervention) vs 8% (control) with 80% power at 2-sided α = .05 in a 20% subsample with a randomized sample of 1300 individuals per group (2600 total) under an intention-to-treat (ITT) convention of counting nonresponders as still vaping. Results are reported according to the Consolidated Standards of Reporting Trials (CONSORT) reporting guideline. The study was conducted by Truth Initiative and approved by the Advarra institutional review board.

### Recruitment, Enrollment, and Randomization

Participant eligibility criteria were age (18-24 years old), current e-cigarette use (past 30 days), interest in quitting vaping in the next 30 days, mobile phone ownership with active text message plan, and US residence. Advertisements on Facebook and Twitter promoted a study on vaping cessation and linked to the study website. Interested individuals completed online eligibility screening. A link to online informed consent was sent via email, thus requiring a valid email for study enrollment. Acceptance of informed consent launched the baseline assessment. Those who completed the baseline were instructed to text a study telephone number. Only those who responded to the system-generated confirmation message within 24 hours were randomized to treatment or control by a computer algorithm that automated random allocation in a 1:1 sequence. Random assignments were concealed from participants and research staff throughout the trial.

### Interventions

To minimize differential attrition and optimize follow-up completion rates, incentivized text message assessments regarding e-cigarette use and abstinence were sent to all participants at 14 days postrandomization and monthly thereafter through a 6-month period. The 14-day query asked, “Checking in: Have you cut down how much you vape nicotine in the past 2 weeks? Respond w/letter: A = I still use the same amount, B = I use less, C = I don't use at all anymore.” The monthly query asked, “How’s the quit going? When was the last time you vaped nicotine, even a puff of someone else’s? Respond w/ letter: A = in the past 7 days, B = 8-30 days ago, C = More than 30 days ago.” All participants were compensated $5 via digital gift card per response (7 assessments total for a maximum compensation of $35). These assessments were designed solely to maximize retention; they were not analyzed as outcomes.

#### This is Quitting

This is Quitting (TIQ) is a fully automated, tailored, interactive text message program for vaping cessation that is designed specifically for young people.^20,21^ It is grounded in best practices from smoking cessation research with young people^17,22,23^ and our experience delivering digital tobacco cessation interventions to people of all ages, informed by formative research with young people. The program is positioned as a nonjudgmental, supportive friend. It is anchored around key constructs from social cognitive theory.^24^ For example, to establish and reinforce perceived social norms and social support for quitting, many messages are written by other users (edited by staff where appropriate). These messages reference the author and convey that many other young people are also quitting (eg, “Ashley says, ‘You can do it we are all in this together.’ You're not the only one who’s thought about quitting.”). To support observational learning, messages include quitting strategies from other young people (eg, “Dalton says, ‘Remember that stress can be dealt with in other ways! Try meditating or even writing down what the problem is and then figure out solutions.’ You dealt with hard things before you started to vape, and you still can.”). To grow behavioral capability, messages give concrete, evidence-based skills and strategies (eg, “Have your friends supported your quitting? Reply YES or NO.” If user responds NO, “Practice—like actually say out loud in front of a mirror at home or in your car—how you’ll turn down a JUUL if they offer it to you.”).

This is Quitting is tailored to a user’s age, enrollment date, or quit date (which can be set and reset via text message), and to the vape product they use (if provided by the user). Those not ready to quit receive 4 weeks of messages focused on building skills and confidence. Users who set a quit date receive messages for 1 week preceding it and 8 weeks afterward that include encouragement and support, skills training and self-efficacy building exercises, coping strategies, and information about the risks of vaping, benefits of quitting, and cutting down to quit. Messages about nicotine replacement therapy describe its use and effectiveness for quitting, its availability over the counter, and that a physician or pharmacist can provide guidance on dosing. Texting keywords such as *TIPS*, *FEELS*, and *STRESS* delivers on-demand support.

This is Quitting is promoted nationally through the truth campaign, the public education campaign run by Truth Initiative for more than 20 years,^25^ and through earned media and local and national outreach efforts. Since it launched in January 2019, more than 300 000 young people (approximately 114 000 teens aged 13-17 years and 186 000 YAs) have enrolled (as of April 2021). To isolate the treatment benefit from any confounding effects related to marketing and to ensure participant blinding, all branding was removed from the program.

#### Assessment-Only Control

After a text message confirming enrollment, participants received only the incentivized text messages asking about e-cigarette use and abstinence as described above. After completing the 7-month follow-up assessment, they were instructed how to enroll in TIQ, if desired.

### Measures

The baseline survey was conducted online and hosted on a secure server. Follow-up assessments at 1 and 7 months postrandomization were conducted via mixed-mode follow-up. Participants who did not complete the survey online were contacted over telephone by research staff blind to treatment assignment. Text messages and emails were used as a final means of gathering abstinence data from nonresponders. Participants were paid $20 for completing each follow-up survey; they earned an additional $10 incentive for responding within 24 hours of the initial invitation.

At baseline, participants provided demographic information (age, gender, race, ethnicity, sexual orientation, subjective financial situation,^26^ student status), frequency of nicotine-containing e-cigarette use (daily or almost daily, less than daily but at least weekly, less than weekly but at least monthly), number of quit attempts in the past year, motivation to quit (“How much do you want to quit vaping? 1 = Not at all, 5 = Very much”), and confidence to quit (“How confident are you that you can quit vaping? 1 = Not at all, 5 = Very much”). Nicotine dependence was assessed with a single item adapted from the Fagerström Test for Nicotine Dependence^19,27^ that asked how soon after waking they vaped. We asked how many of the participant’s 5 closest friends vaped, whether they lived with someone who vapes nicotine, and whether they lived with someone who uses other tobacco products. Measures of other substance use assessed past 30-day use of cigarettes and marijuana/cannabis and past 30-day binge drinking.^28^ The Patient Health Questionnaire-2 (PHQ-2)^29^ and Generalized Anxiety Disorder-2^30^ screened for depression and anxiety, respectively. Scores of 3 or more defined a positive screen.

At 1 month, participants rated their experience with the intervention (eg, “They suggested quitting strategies that were new to me.” “They made me feel less alone in quitting.”) on a 4-point scale (1 = completely agree; 2 = somewhat agree; 3 = somewhat disagree; 4 = completely disagree). The primary outcome was self-reported 30-day abstinence at 7 months postrandomization (“In the past 30 days, did you vape at all, even a puff of someone else’s?”); 7-day abstinence was assessed similarly. Participants were instructed to consider use of all nicotine-containing vaping devices (including JUUL, mods, and other e-cigarettes) when answering these questions.

### Statistical Analysis

Primary outcome analyses compared 30-day point prevalence abstinence (ppa) at 7 months postrandomization in study arms using the glm function in R software, version 4.0.2 (R Foundation for Statistical Computing). As described in the study protocol,^20^ we first conducted an ITT analysis in which participants lost to follow-up were assumed to be treatment failures (ie, vaping). Sensitivity of the findings to the missing = vaping assumption was assessed via a multiple imputation model, in which the unknown association between loss to follow-up and vaping abstinence was varied over a very broad range of possible values.^31^ As an alternative to ITT analyses, inverse probability of retention weighting (IPRW) was used to correct observed outcomes for participants’ differential propensity to provide 30-day abstinence data.^32^ We estimated the response rate in each arm conditionally on baseline characteristics presented in Table 1, divided these propensity scores by response rates in each arm, and inverted the resulting ratios to create stabilized weights of unit mean under the assumption of no model misspecification. We assessed the success of the IPRW approach in reducing selection bias due to nonresponse by comparing standardized mean differences (SMD) in baseline characteristics between respondents and nonrespondents.^33,34,35^ An SMD of 0.2 pooled SDs after weighting was used as an indication that the propensity-weighting procedure was successful in balancing an individual characteristic.^36^ Stabilized weights were used to estimate logistic regression models for between-arm differences in abstinence outcomes via the survey package in R software, version 4.0.2. No additional covariates were employed in either ITT nor IPRW outcome models.

**Table 1.** Self-reported Baseline Characteristics of Enrolled Participants

Characteristic	Total (N = 2588)	Control arm (n = 1284)	Intervention arm (n = 1304)
Age, mean (SD), y	20.4 (1.7)	20.4 (1.7)	20.4 (1.7)
Gender			
Female	1303 (50.3)	652 (50.8)	651 (49.9)
Male	1253 (48.4)	619 (48.2)	634 (48.6)
Nonbinary or other	26 (1.0)	8 (0.6)	18 (1.4)
Refused	6 (0.2)	5 (0.4)	1 (0.1)
Race			
White	2159 (83.4)	1067 (83.1)	1092 (83.7)
Asian	123 (4.8)	52 (4.0)	71 (5.4)
Black	38 (1.5)	19 (1.5)	19 (1.5)
American Indian/Alaska native	18 (0.7)	7 (0.5)	11 (0.8)
Multiracial	162 (6.3)	90 (7.0)	72 (5.5)
Other	50 (1.9)	25 (1.9)	25 (1.9)
Refused	28 (1.1)	24 (1.9)	14 (1.1)
Hispanic ethnicity	275 (10.6)	134 (10.4)	141 (10.8)
Sexual minority	493 (19.0)	250 (19.5)	243 (18.6)
Income			
Lives comfortably	673 (26.0)	349 (27.2)	324 (24.8)
Meets needs with a little left	1000 (38.6)	495 (38.6)	505 (38.7)
Just meets basic expenses	778 (30.1)	379 (29.5)	399 (30.6)
Does not meet basic expenses	137 (5.3)	61 (4.8)	76 (5.8)
Current student	1932 (74.7)	956 (74.5)	976 (74.8)
Nicotine vaping frequency			
Daily or almost daily	2410 (93.1)	1189 (92.6)	1221 (93.6)
Less than daily but at least weekly	145 (5.6)	73 (5.7)	72 (5.5)
Less than weekly but at least monthly	33 (1.3)	22 (1.7)	11 (0.8)
Time to first vape after waking			
Within 30 min	2129 (82.3)	1045 (81.4)	1084 (83.1)
After 30 min	458 (17.7)	238 (18.6)	220 (16.9)
Attempts to quit vaping in past year			
None	222 (8.6)	110 (8.6)	112 (8.6)
1-2 times	674 (26.0)	327 (25.5)	347 (26.6)
3-5 times	911 (35.2)	472 (36.8)	439 (33.7)
≥6 times	781 (30.2)	375 (29.2)	406 (31.1)
Motivation to quit vaping (score range, 1-5), mean (SD)	4.54 (0.70)	4.55 (0.69)	4.53 (0.71)
Confidence to quit vaping (score range, 1-5), mean (SD)	3.47 (1.15)	3.49 (1.17)	3.44 (1.14)
No. closest friends that vape nicotine, mean (SD)	2.91 (1.49)	2.89 (1.50)	2.94 (1.49)
Live with e-cigarette (nicotine) user	1165 (45.0)	588 (45.8)	577 (44.2)
Live with tobacco user	916 (35.4)	461 (35.9)	455 (34.9)
Use of cigarettes in past 30 d	841 (32.5)	403 (31.4)	438 (33.6)
Use of marijuana/cannabis in past 30 d	1534 (59.3)	733 (57.1)	801 (61.4)
Binge drinking in past 30 d	1929 (74.5)	959 (74.7)	970 (74.4)
PHQ-2 score ≥3	910 (35.2)	445 (34.7)	465 (35.7)
GAD-2 score ≥3	1134 (43.8)	562 (43.8)	572 (43.9)

Abbreviations: GAD-2, Generalized Anxiety Disorder-2; PHQ-2, Patient Health Questionnaire-2.

To identify potential moderators of the treatment-outcome relationship, we examined interactions between treatment assignment and variables presented in Table 1 (details of these analyses are outlined in eAppendix 1 in Supplement 2). We also conducted stratified outcome analyses by level of nicotine dependence (vape within 30 minutes after waking vs not) to assess whether treatment effects persisted among those with higher dependence levels. All hypothesis tests were conducted at a 2-tailed α = .05 significance level.

## Results

Between December 29, 2019, and March 18, 2020, 11 080 individuals were screened for eligibility and 2588 participants were randomized (1284 to the control arm and 1304 to TIQ). At 1 month, the response rate was 79.5% (n = 2057 of 2588), with slightly higher retention rates in the control arm vs TIQ (81.4% [n = 1045 of 1284] vs 77.6% [n = 1012 of 1304], respectively; *P* = .02; SMD = 0.09). At 7 months, the response rate was 76.0% (n = 1967 of 2588), with no difference between the control and TIQ arms (77.4% [n = 994 of 1284] vs 74.6% [n = 973 of 1304], respectively; *P* = .11; SMD = 0.06; Figure).

**Figure.**
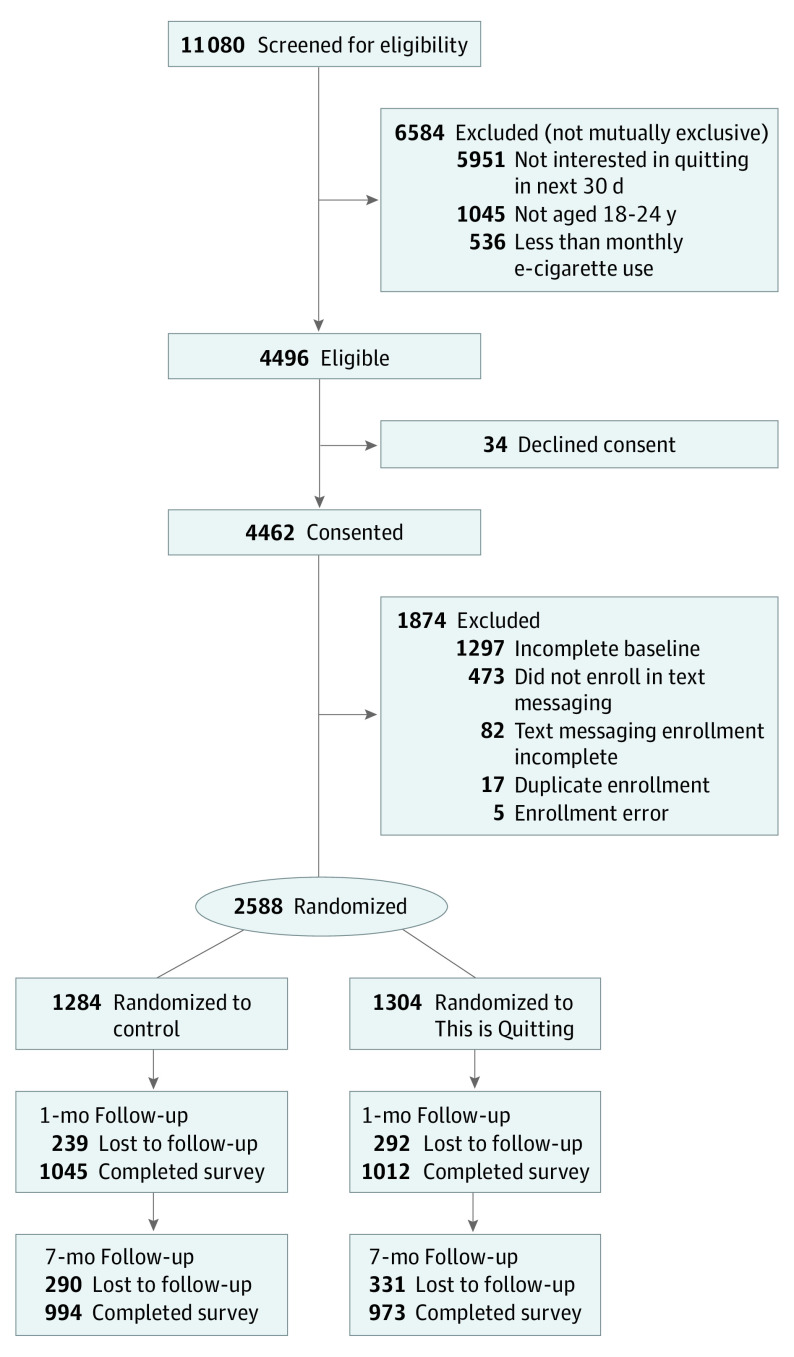
CONSORT Diagram

The mean (SD) age of the 2588 participants was 20.4 (1.7) years (Table 1). Of the total sample, 1253 (48.4%) were male, 2159 (83.4%) were White, 275 (10.6%) were Hispanic, and 493 (19.0%) were a sexual minority. Roughly one-third (n = 915 [35.4%]) reported barely or not meeting basic expenses. Three-quarters of the sample (n = 1932 [74.7%]) were current students. The majority of participants (n = 2410 [93.1%]) vaped nicotine daily, and 2129 (82.3%) reported vaping within 30 minutes of waking. Participants reported a strong desire to quit vaping (mean [SD], 4.54 [0.70]) but were less confident about their ability to quit (mean [SD], 3.47 [1.15]). Most (n = 2366 [91.4%]) had tried to quit in the past year, and 1692 (65.4%) had made 3 or more quit attempts. Past 30-day use of other substances was as follows: cigarettes, 32.5% (n = 841); marijuana/cannabis, 59.3% (n = 1534); and binge drinking, 74.5% (n = 1929). More than one-third (n = 910 [35.2%]) had a positive screen for depression on the PHQ-2, and 1134 (43.8%) had a positive screen for anxiety on the Generalized Anxiety Disorder-2; 1317 (50.9%) had a positive screen on either measure. No between-arm differences exceeded an SMD of 0.2, indicating a balanced sample with no clinically meaningful differences between arms.

### Vaping Cessation

As shown in Table 2, 30-day ppa rates at 7 months under ITT were 24.1% (n = 314 of 1304) among TIQ participants and 18.6% (n = 239 of 1284) among controls. A comparison of baseline characteristics between 7-month responders and nonresponders showed that race (SMD = 0.25) and student status (SMD = 0.26) exceeded the threshold for a small effect (eTable 1 in Supplement 2). A multivariate logistic regression model with 7-month response status as the outcome identified Hispanic ethnicity, sexual minority status, current student status, and PHQ-2 score as predictors of study retention, with current student status and PHQ-2 score resulting in nondifferential attrition by study arm. The IPRW analyses of 7-month outcomes showed that the treatment benefit owing to TIQ in the odds ratio (OR) scale was broadly similar under ITT (OR, 1.39; 95% CI, 1.15-1.68; *P* < .001) and IPRW (OR, 1.51; 95% CI, 1.24-1.85; *P* < .001) analyses. Table 2 also shows 7-day ppa under IPRW and ITT analyses; TIQ yielded higher quit rates than control under both analytic approaches, though effect sizes were smaller than those for 30-day ppa. Sensitivity analyses based on multiple imputation modeling confirmed the robustness of ITT estimates (eAppendix 2 and eTable 2 in Supplement 2).

**Table 2.** Vaping Cessation Outcomes Under Intention-to-Treat and Complete-Case Analyses at 7 Months

Outcome variable	% (95% CI)		Rate difference (95% CI)	Rate ratio (95% CI)	Odds ratio (95% CI)	*P* value
	Control arm (n = 1284)	Intervention arm (n = 1304)				
7-d ppa						
No. of responses	994	973	NA	NA	NA	NA
No. abstinent	385	440	NA	NA	NA	NA
Intention to treat	30.0 (27.5-32.6)	33.7 (31.2-36.4)	3.8 (0.2-7.3)	1.13 (1.01-1.26)	1.19 (1.01-1.40)	.04
IPRW outcomes	40.0 (37.0-43.3)	45.8 (42.7-49.1)	5.8 (1.3-10.3)	1.14 (1.03-1.27)	1.27 (1.05-1.52)	.01
30-d ppa						
No. of responses	994	973	NA	NA	NA	NA
No. abstinent	239	314	NA	NA	NA	NA
Intention to treat	18.6 (16.7-20.8)	24.1 (21.8-26.5)	5.5 (2.3-8.6)	1.29 (1.11-1.50)	1.39 (1.15-1.68)	<.001
IPRW outcomes	24.0 (21.3-26.7)	32.3 (29.3-35.3)	8.2 (4.3-12.2)	1.35 (1.16-1.56)	1.51 (1.24-1.85)	<.001

Abbreviations: IPRW, inverse probability of retention weighting; NA, not applicable; ppa, point prevalence abstinence.

### Moderator Results

Analyses of all variables in Table 1 as potential moderators of treatment effects on 30-day ppa rates under both ITT and IPRW analysis yielded no statistically significant findings (eTable 3 in Supplement 2). In stratified analyses focused on time to first vape, ITT abstinence rates among those who reported vaping within 30 minutes of waking were slightly lower in both study arms but still favored TIQ over control (22.6% vs 16.4%; *P* < .001). Among those who reported vaping 30 minutes after waking, ITT abstinence rates were higher in both study arms with no difference between TIQ and control (31.4% vs 28.6%; *P* = .51).

### Intervention Satisfaction

As shown in Table 3, TIQ participants reported higher levels of intervention satisfaction compared with control participants across all items. The largest effect size (SMD = 0.668) was observed for the item “They suggested quitting strategies that were new to me.” Three items were of small-moderate magnitude: (1) “They made me feel that I knew the right steps to take to quit” (SMD = 0.347), (2) “They made me feel less alone in quitting” (SMD = 0.344), and (3) “They helped me feel more confident about quitting” (SMD = 0.224).

**Table 3.** This is Quitting Intervention Satisfaction at 1-Mo Follow-up^a^

Survey item	Score, mean (SD)^b^		*P* value	SMD
	Control arm	Intervention arm		
They suggested quitting strategies that were new to me	2.63 (0.94)	2.03 (0.85)	<.001	0.668
They made me feel that I knew the right steps to take to quit	2.23 (0.94)	1.93 (0.78)	<.001	0.347
They made me feel less alone in quitting	2.08 (0.92)	1.78 (0.78)	<.001	0.344
They helped me feel more confident about quitting	1.99 (0.86)	1.81 (0.75)	<.001	0.224
They helped me stay on track with quitting	2.20 (0.80)	2.05 (0.71)	<.001	0.197
They were written personally for me	2.73 (0.88)	2.59 (0.86)	<.001	0.162
I liked being able to interact with the text messages	1.85 (0.80)	1.72 (0.75)	<.001	0.161

Abbreviation: SMD, standard mean difference.

^a^ Lower scores indicate more positive response.

^b^ Participants responded to items on the following scale: 1 = Completely agree; 2 = Somewhat agree; 3 = Somewhat disagree; 4 = Completely disagree.

## Discussion

Results of this randomized clinical trial demonstrated the effectiveness of a tailored, interactive text message intervention for vaping cessation among YAs compared with an assessment-only control. Participants randomized to TIQ were one-third more likely to quit vaping at the 7-month primary end point compared with control participants. Estimates of the treatment benefit appear robust to assumptions about missing data, as response rates were similar in both arms. Furthermore, the superiority of the intervention was consistent across all demographic variables and vaping characteristics examined, including nicotine dependence, social influences to vape, and other substance use.

The high absolute magnitude of quit rates in both arms is encouraging and worthy of further exploration. Although, to our knowledge, there are no studies of vaping cessation interventions available for comparison, smoking cessation interventions among young people have generally performed less well.^18^ Recruitment during “quitting season” (ie, the weeks leading up to and including New Year’s Day) may have resulted in higher motivation to quit and quit rates in both arms than if recruitment had been conducted at other times during the year, though previous research with adult smokers has countered this hypothesis.^37^ Additionally, incentivized text message assessments may have resulted in assessment reactivity in both arms. Finally, the trial was conducted during the unprecedented social disruption of the COVID-19 pandemic, which may have affected quit rates in a variety of ways.^38,39^

### Strengths and Limitations

Strengths of this study include a large and diverse sample across a number of demographic characteristics (race, sexual orientation, income) that was representative of the population from which it was drawn.^40^ Follow-up rates (75%-77%) were higher than those in many smoking cessation studies conducted among YAs and were gathered at a longer follow-up interval.^18^ There was no differential attrition at 7 months despite the use of an assessment-only control.

Two potential limitations are worth noting. We did not conduct biochemical verification of abstinence given the demonstrated challenges in digital cessation studies,^41,42^ the lack of demand characteristics that would give rise to misreporting,^43,44^ and the selection of 30-day abstinence as a more rigorous primary end point. Second, this study did not include teens, in whom rates of e-cigarette use are highest. To date, more than 114 000 13- to 17-year-olds have enrolled in TIQ, demonstrating the appeal of this approach among teens. Future research should evaluate its effectiveness in this age group.

## Conclusions

This randomized clinical trial demonstrated the effectiveness of a tailored, interactive text message intervention in promoting vaping cessation among YAs. Text messaging is a scalable and cost-efficient approach to delivering vaping cessation treatment on a population basis. These results establish a benchmark of effectiveness for other vaping cessation programs and begin to fill an important gap in understanding how to help young people quit e-cigarettes.

## Notes

Supplement 1. Trial Protocol

Supplement 2. eAppendix 1. Moderation Analyses eAppendix 2. Multiple Imputation Approach eTable 1. Comparison of Baseline Characteristics Between Non-Responders and Responders at 7-Month Follow-Up eTable 2. Sensitivity of Intervention Effects to Missing Data Assumptions eTable 3. Moderators of Intervention Effects on 30-day ITT Abstinence eReferences

Supplement 3. Data Sharing Statement.
