# Supplementary material for: Effectiveness of a Vaping Cessation Text Message Program Among Young Adult e-Cigarette Users: A Randomized Clinical Trial
Source: JAMA Intern Med. 2021 May 17;181(7):923–30. doi: 10.1001/jamainternmed.2021.1793 (PMC8129897; doi:10.1001/jamainternmed.2021.1793)
Supplement: Supplement 1. — Trial Protocol [file jamainternmed-e211793-s001.pdf]

RESEARCH PROTOCOL

Protocol Title and Number:
A randomized trial of a text message quit vaping intervention for young adults

Date of Submission to Advarra:
November 22, 2019

Sponsor:
Truth Initiative
900 G Street, NW
Fourth Floor
Washington, DC 20001

Principal Investigator:
Amanda L. Graham, PhD
Chief, Innovations
Truth Initiative
900 G Street, NW
Fourth Floor
Washington, DC 20001
T: 202/454-5938
agraham@truthinitiative.org

Final Signed Protocol:

Amanda L. Graham, PhD
Principal Investigator Name

Amanda L Graham
Principal Investigator Signature

November 22, 2019
Date

BACKGROUND

The popularity of electronic cigarettes (or e-cigarettes) has grown exponentially over the past few years, with the surgeon general calling the use of e-cigarettes among young people an "epidemic".[1] E-cigarettes are now the most commonly used tobacco product among youth and in the U.S., youth and young adults are more likely than adults to use e-cigarettes. Many younger e-cigarette users have never used another tobacco product: among current e-cigarette users aged 45 years and older in 2015, most were either current or former regular cigarette smokers; in contrast, among current e-cigarette users aged 18-24 years, 40.0% had never been regular cigarette smokers.[2] E-cigarette use has grown even faster in recent years due to the popularity of products such as JUUL, a small-USB like e-cigarette with high nicotine content that is easy to conceal. A 2018 study by our colleagues at Truth Initiative showed that the highest percentage of e-cigarette users were ages 18-21 years old.[3]

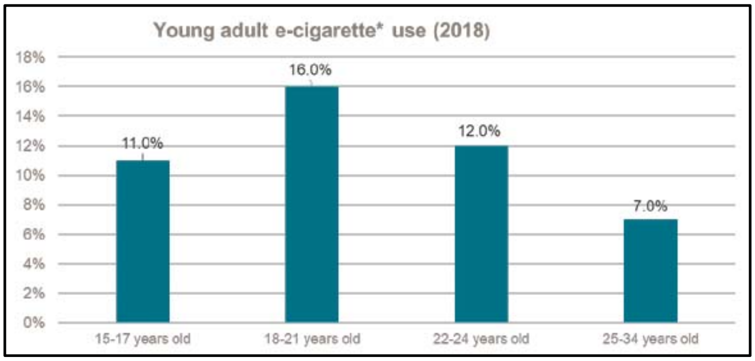

Young people should never use nicotine in any form. While there is some evidence that e-cigarettes are less harmful than traditional cigarettes, they are not harmless and early exposure to nicotine can have long-lasting adverse effects. The majority of e-cigarettes contain nicotine, which has known health effects on brain development which occurs into the mid-20s. Specific risks include nicotine addiction, mood disorders, permanent lowering of impulse control, as well as negative impacts on attention and learning. The aerosol produced by e-cigarettes contains cancer-causing chemicals and tiny particles that reach deep into the lungs. E-cigarettes have also produced unintended injuries such as fires and explosions from defective batteries and poisonings from acute nicotine exposure through e-cigarette liquids. Finally, there is strong evidence that e-cigarette use can lead to the use of other tobacco products such as cigarettes.[4,5] Reversing the dramatic gains over the past decade in reducing cigarette smoking among young people would be nothing short of a public health tragedy.

In response to this epidemic and given no available quitting resources for young people (cessation resources such as medication have no demonstrated effectiveness for young people[6,7]), Truth Initiative launched a first-of-its kind e-cigarette cessation program designed specifically for young people in January 2019. The program is called *This is Quitting* and it is delivered entirely via text message. Mobile phone ownership is ubiquitous among young people and text messaging is a preferred communication modality in this age group. Text messaging is also easy to use, discreet, anonymous, and there is strong evidence supporting its effectiveness as a cessation intervention modality.[8] *This is Quitting* was developed using best practices from smoking cessation research with young people, our extensive experience delivering digital tobacco cessation interventions to people of all ages, as well as formative research with young e-cigarette users and quitters.

Young people enroll by texting "DITCHJUUL" to a short code and responding to the initial message with their age and first name (used for tailoring the program). Terms of Service and Privacy Policy are provided via a link in a text message (included in Appendix A). Users receive one age-appropriate message per day tailored to their enrollment date or quit date, which can be set and reset via text message. Those not ready to quit receive 4 weeks of messages focused on building skills and confidence. Users who set a quit date receive messages for a week preceding it and 8 weeks afterward that include encouragement and support, skill- and self-efficacy building exercises, coping strategies, and information about the risks of vaping, benefits of quitting, and cutting down to quit. Keywords COPE, STRESS, SLIP, and MORE provide on-demand support. Sample text messages can be found at the end of this protocol. Users can unsubscribe anytime by texting STOP. E-cigarette use and abstinence are assessed via text message at 14, 30, 60, and 90 days following an enrollee's quit date or enrollment date. At 14 days, enrollees are asked, "Have you cut down how much you JUUL in the past 2 weeks? Respond w/letter: A=I still JUUL the same amount, B=I JUUL less, C=I don't JUUL at all anymore." At 30, 60, and 90 days, enrollees are asked, "When was the last time you JUULed, even a puff of someone else's? Respond w/ letter: A: in the past 7 days, B: 8–30 days ago, C: More than 30 days ago."

To date, more than **58,000 young people have enrolled**, demonstrating the appeal of this treatment approach and the urgent need for quit vaping resources among young people. Preliminary evaluation data collected among an initial cohort of roughly 27,000 users has shown high levels of engagement in the program and high rates of abstinence among responders.[9] Roughly ¾ of users set a quit date, with the most common quit date being the day of enrollment. Interactive keywords were used by 45.5% of teens and 38.4% of YA. Response rates to follow-up assessment questions were 36.9% at 14 days and 21.0% at 90 days. At 14 days, 60.8% of respondents indicated they had reduced or stopped using e-cigarettes altogether. At 90 days, 7-day point prevalence abstinence (ppa) was 25% and 30-day ppa was 16%. The high volume of enrollment in a short period of time, high levels of

engagement with the program, and e-cigarette reduction and cessation results demonstrate that young people are interested in quitting vaping and can be engaged in an easily accessible, anonymous digital platform.

Building on this preliminary evidence, the primary aim of the current study is to conduct a fully powered comparative effectiveness trial to evaluate the effectiveness of *This is Quitting* in promoting abstinence from e-cigarettes among young users aged 18-24. This study is a 2-arm randomized controlled trial conducted among young users aged 18-24 recruited through online channels. Participants will be randomized to *This is Quitting* or to an assessment-only control condition and followed for 7 months to roughly correspond to 6-months post-treatment. The secondary aim is to examine potential mediators of program effectiveness, including treatment engagement and changes in self-efficacy and perceived social support for quitting.

The scientific, clinical, and public health communities are desperate for proven, evidence-based cessation resources to address the vaping epidemic among young people. We are uniquely positioned to address this urgent need given our extensive experience in conducting large-scale, rigorous research trials of digital tobacco cessation interventions and our expertise in working with young people. We plan to rapidly disseminate study results to benefit other researchers/service providers and use these results to further enhance the program.

METHODS

Subjects

We will recruit N=1720 e-cigarette users aged 18-24 years old who are interested in quitting in the next 30 days. Other eligibility criteria include past 30-day e-cigarette use and US residence. To fully enroll in the study, individuals must sign up for *This is Quitting* and respond to the initial system-generated message.

Recruitment and Enrollment

Potential participants will respond to online ads on various platforms (Facebook, YouTube, Instagram, Reddit, etc.) if they are current e-cigarette users who are interested in quitting and willing to participate in a research study. Clicking on the ad will lead them to a webpage that provides details about what participation in the study entails and describes incentives for participation. Those who are interested will complete an eligibility screener followed by informed consent and a baseline assessment. Those who complete the baseline will be randomized into one of two arms (see below for additional details) and instructed to text a specific keyword to the phone number corresponding to their treatment assignment. Only those who respond to the first message from the text message program within 24 hours will be fully enrolled into the study. This requirement will be made explicit.

Randomization

Randomization occurs after completion of the online enrollment process (completed baseline survey). A computer algorithm that is part of the survey software will automate random allocation.

Interventions

This is Quitting: Participants will be enrolled to receive messages from This is Quitting as described above. ***Assessment-only control.*** After an initial enrollment message, participants will be contacted periodically to assess e-cigarette use. At the end of the intervention period, they will receive information on how to sign up for This is Quitting if they are interested in the program (optional).

Potential Problems and Solutions

Loss to follow-up is the primary concern in trials involving digital interventions, especially ones that employ an assessment only control. We expect at least 75% follow-up at 1-month and 65% at 7-months based on our previous and ongoing digital mobile-based evaluations. If follow-up rates are lower than expected early in the trial, we will consider shortening the 7-month follow-up to gather only abstinence outcomes. To maximize follow up rates we will:

1) provide clear information about the study at the outset, including expectations for follow-up;
2) reimburse participants up to $30 per follow-up assessment;
3) collect interim abstinence assessments via text ($5 incentive for each response) for both arms to optimize the likelihood that participants randomized to the assessment-only control stay engaged with the study;
4) send reminders about follow-up surveys via email and text messages;
5) conduct phone follow-ups for those unreachable by email and text messages; and,

6) emphasize the importance of survey completion regardless of abstinence status.

Measures

All randomized participants in both arms will be asked to complete all assessments. Assessments will occur at baseline and 1 month and 7 months post-randomization. The baseline survey will be conducted online and hosted on a secure server. Mixed-mode follow-up (email, phone, text) will be employed. Telephone surveys will be conducted by research staff blind to treatment. Text messages have demonstrated moderately high reliability (k=.66) compared with web-based surveys in assessing smoking outcomes[10] and will be used as a final means of gathering abstinence data from non-responders. Most measures listed below are standard instruments used in cessation studies, and are reliable when administered via the Internet.[11,12]

Screening Variables. To characterize the sample of users interested in the study and assess for eligibility, we will gather: *demographics* (age, education, income level, sexual and gender identity, race, and ethnicity); *current e-cigarette use* (use of e-cigarette containing nicotine or THC in the past 30 days)[13]; *interest in quitting*; *contact information* (e-mail and phone number to send baseline survey to and link text message sign-up data). These questions are being asked up front so we can determine whether users enrolling in the study are representative of the wider population of e-cigarette users.

Baseline Variables. To characterize the sample and explore potential moderators of treatment effectiveness, we will gather: additional *demographics* (student status, employment status); *current e-cigarette use and history* (frequency and rate,[13,14] motivation to quit and quitting history[15]); *nicotine dependence* will be assessed with the PROMISE-E[16] and items from the Texas Adolescent and Tobacco Marketing Surveillance[17]; *other substance use* (other tobacco products, alcohol)[18] and *mental health symptoms*.[19] Given evolving trends of e-cigarette use and perception, we will also ask about their awareness about media reports about e-cigarettes, perception about e-cigarettes, reasons for wanting to quit, and reasons for joining the study. To account for potential predictors of dropout, we will ask about motivation to use or quit e-cigarettes and potential barriers to quitting (e.g., social influences). We will assess baseline levels of perceived level of social support, perceived social norms, and perceived self-efficacy for quitting and examine changes in these variables as potential mediators of treatment effectiveness.

Mediating Variables. We hypothesize that treatment engagement will mediate the relationship between treatment assignment and abstinence outcomes. We will extract data regarding replies to interactive text messages (e.g., setting a quit date, keyword use) as well as unsubscribe status, number of days enrolled, and total number of messages received. All text message interactions are date/time stamped.

Outcome Measures. The *primary outcome* is self-reported 30-day point-prevalence abstinence (ppa) at 7-months but we will gather abstinence data at all follow-ups. *Other quitting-related outcomes* include change in motivation to quit, quit attempts, reduction in e-cigarette use, 7-day ppa, and continuous abstinence measured at each formal follow-up as well as interim text message assessments (single item asking about current vaping status). *Intervention satisfaction* in both conditions will be measured with items about overall satisfaction and whether they would recommend it to a friend (scale from 0-10). Satisfaction with frequency of text messages will be measured.[20] To assess *perceived message relevance*, participants will be asked whether text messages "were written personally for you"[21] and "were directed at you personally".[22]

Data Analysis Plan

Primary Analysis. Point-prevalence abstinence (ppa) at 6-months post-treatment will be compared across the treatment and control groups using logistic regression. All estimates will be adjusted for baseline confounders of the intervention-outcome relationship. We will identify potential moderators (e.g., age, gender, baseline motivation to quit) by analyzing interactions between treatment and selected variables. For all moderators found to be associated with the primary outcome, we will examine the effects of treatment/moderator interaction terms on outcomes after entering main effects.

Secondary Analyses. Additional outcomes related to abstinence and treatment engagement will also be analyzed with logistic regression as secondary analyses. These include likelihood of making a quit attempt, likelihood of reducing e-cigarette use, and changes in confidence and self-efficacy in quitting e-cigarettes.

Missing Data. Missing data will be handled in two ways. First, we will conduct an intent-to-treat (ITT) analysis in which participants who have been lost to follow up are assumed to be treatment failures (i.e., vaping). This analysis will be conducted because ITT analyses are common in the smoking cessation literature, despite simulations demonstrating that the approach is neither conservative nor anti-conservative but rather biased in favor of whichever condition contains less missingness.[23] Second, we will supplement the ITT analyses with an analysis that uses a multiple imputation (MI) procedure to minimize bias in estimates and standard errors, under the assumption that outcomes are not missing at random (NMAR) but rather more likely to be missing for treatment failures (i.e., vaping) than treatment successes (i.e. abstinence). Since the magnitude of actual response bias is unknown, we will conduct a sensitivity analysis to evaluate the treatment effect on outcomes under a range of magnitudes, from equal odds of missing (OR=1) to five times more likely to be missing (OR=5).

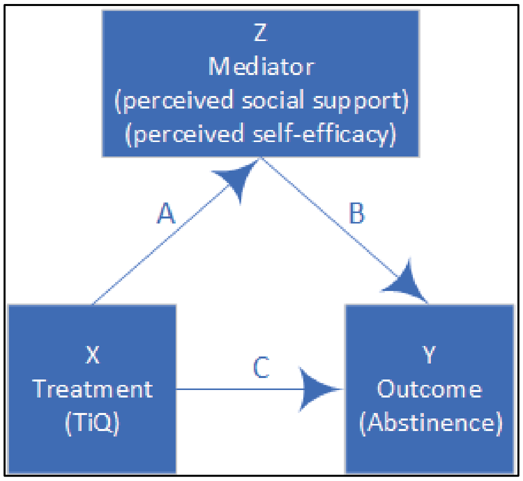

Mediator Analysis. Our conceptual model is that treatment increases the odds of abstinence by increasing perceived social support and perceived self-efficacy for quitting. Those two constructs will be measured at baseline, 1-month and 7-months post-treatment. Change in those constructs from baseline will be evaluated with separate mediation analyses as presented in Figure 1. Specifically, we hypothesize that the effect of treatment on abstinence will be mediated by perceived social support and perceived self-efficacy, such that: (1) a significant effect A is found associating X with Z ; (2) a significant effect B is found associating Z with Y; (3) a significant effect C is found associating X with Y; and (4) the effect of C is significantly attenuated when A and B are simultaneously included in the model.

PROTECTION OF HUMAN SUBJECTS

Human Subjects Involvement, Characteristics, and Design

Participants will be 1,720 e-cigarette users aged 18-24 who respond to online advertising about a research study and who complete enrollment into a text message program based on random allocation. Study enrollment is conducted entirely online and is fully automated. Randomization occurs after participants have provided informed consent and completed the baseline survey. Study enrollment will not be considered complete until the participant responds to the initial system-generated text message. There will be no involvement of vulnerable populations and no deception. Children (under the age of 18) will be excluded.

Sources of Materials

Sources of research material include the following: 1) screening data, 2) text message utilization data, and 3) baseline and follow-up assessments. The baseline assessment will be conducted online with the survey hosted on a secure server. Mixed-mode follow-up (online, phone, text message) will be employed. Phone surveys will be conducted by research staff blind to treatment condition.

Materials Access

For all data, the Principal Investigator (Dr. Amanda Graham), Data Analyst (Dr. Michael Amato), Project Managers (Sarah Cha, Megan Jacobs), research assistants (Mia Bottcher, Jenna Woodward), and the software development team will have access to individually identifiable information about human subjects. Electronic data files with identifiable information will be maintained separately from other data files and will only be used for administrative purposes (e.g., tracking follow-up completion, managing subject payment). All personnel already have or will have received certification in human subjects protection prior to beginning work on this project.

Potential Risks

The overall risk is judged to be very low. Study participants who attempt to quit e-cigarette use will likely experience some nicotine withdrawal symptoms that may include anxiety, restlessness, anger, irritability, sadness, and problems concentrating. There is no reason to believe that participation in this study would worsen nicotine withdrawal symptoms or that symptoms would differ based on randomization assignment.

ADEQUACY OF PROTECTION AGAINST RISKS

Enrollment and Informed Consent

Step 1 (Study information): Potential participants who click on an online ad for e-cigarette users who are interested in quitting and willing to participate in a research study will be directed to a website that provide additional details about the study. This webpage will provide information on who is behind the study, what participation entails, and who may be eligible for the study. **Step 2 (Eligibility screening):** Individuals who are interested in participating will complete a short survey to confirm eligibility for study participation. Those who are eligible will be asked to provide contact information (e-mail address to send links to surveys and mobile number for text message sign up). **Step 3 (E-mail confirmation):** An email message is sent to the participant with a distinct URL (Web address) containing the baseline survey and instructions to complete the survey within 24 hours to complete study enrollment. This process ensures that enrolled participants have a valid email address where they can receive study-related correspondence. **Step 4 (Informed consent and baseline assessment):** When the participant clicks on the URL in the email, it will open up an online baseline survey. On the first page of the baseline survey, a full informed consent text will be provided, including a) each study condition; b) financial incentives for participating; c) the process of randomization and the equal chance of being assigned to one of two treatment conditions; d) protection of confidentiality and the right to withdraw at any time; and e) expectations regarding follow-up data collection, including compensation for time required to complete follow-up assessments (regardless of e-cigarette use status). Users will be required to select "Yes, I would like to continue" as active affirmation that they are providing informed consent for study participation. Contact information for the study and the Institutional Review Board where IRB approval has been obtained will be included in the consent form. All information about the study will be written at a 6th grade reading level to ensure adequate comprehension. Agreeing to participate will launch the baseline assessment. **Step 5 (Randomization):** Randomization will only occur after an eligible study participant has completed the online enrollment process (confirmed eligibility, indicated informed consent, and completed baseline survey). **Step 6 (Text message sign-up):** Users will be randomized at the conclusion of the baseline assessment and provided with a phone number to text based on their assignment. Users must text their assigned number and reply to the welcome text message to confirm text message enrollment and finalize their study enrollment.

Protections Against Risk

Exposure to evidence-based information and support for e-cigarette cessation in the intervention arm is expected to attenuate withdrawal symptoms associated with e-cigarette cessation that may occur.

POTENTIAL BENEFITS OF THE PROPOSED RESEARCH TO HUMAN SUBJECTS AND OTHERS

Subjects may benefit from participation in this research in several ways. By participating in an e-cigarette cessation treatment program, subjects may increase the likelihood that they will attempt to quit e-cigarette use and be successful. Alternatively, participants will gain a greater understanding of their e-cigarette use behavior that may help them to quit successfully in the future. Finally, although this is not a direct benefit, all participants will be involved in a project to evaluate innovative treatments for e-cigarette cessation that may ultimately produce an enormous public health impact. We believe the substantial potential benefits significantly outweigh the minimal potential risks to research participants.

Payment for participation

There is no payment for study enrollment or completion of the baseline assessment. All participants in both arms are asked to complete follow-up surveys at 1- month and 7-months post-randomization. Participants will be paid $20 for completing each follow-up survey via the Internet or over the phone with a telephone interviewer. Participants who respond within 24 hours of receiving the initial survey invitation will receive an additional $10. All participants in both arms will also be compensated $5 for each text message assessment (7 total) they respond to, for a total compensation of up to $95 for their entire study participation.

Payment for completing the surveys will be delivered via Rybbon, a company specializing in digital gift management for marketing and research. Rybbon is fully integrated with Qualtrics. When a participant completes a follow-up survey via Qualtrics, Rybbon will generate a unique link for giftcard redemption and will email it to the participant. Rybbon has no access to any other identifying information other than email and no access to survey data. Participants can redeem their Rybbon giftcard from a variety of giftcard options. Those completing text message assessments will receive a link to a Rybbon payment via text message upon responding to each assessment.

IMPORTANCE OF THE KNOWLEDGE TO BE GAINED

This is Quitting is the first text message intervention for e-cigarette cessation designed specifically for young people. We have seen a high volume of enrollment since the program launched in January 2019 with only earned media and organic marketing efforts to date. We have been flooded with interest from youth-serving organizations looking for a proven quit vaping program to offer to their young people. To our knowledge, this study will be the first to rigorously evaluate a scalable, cost-efficient, market-tested quit vaping program among users aged 18-24. These data are urgently needed.

DATA AND SAFETY MONITORING PLAN

Confidentiality will be protected at all times and potential risks will be minimized systematically.

Data Safety

Respondents will fill out computer-based surveys. All data collected via surveys will be stored in a secure Qualtrics database online (see **Appendices A & B** for information on data security and storage). All research studies and their related non-public materials are confidential information. This includes, but is not limited to, the study title, study questions that are responded to, provided as part of a study, and any concepts related to those materials. Respondent information will remain confidential.

All data used specifically for this project will be maintained in a manner consistent with NIH standards. The Principal Investigator (Dr. Amanda Graham), Data Analyst (Dr. Michael Amato), Project Managers (Sarah Cha, Megan Jacobs), research assistants (Mia Bottcher, Jenna Woodward), and software development team will have access to participants' identifying information. All study staff will have received certification in human subjects protection from the NIH Office of Human Subjects Research. Confidentiality of data will be maintained by numerically coding all data, by disguising identifying information, and by keeping all data electronically protected. Electronic data files with identifiable information will be maintained separately from other data files and will only be used for administrative purposes. Identifying information will not be reported. The list of participant phone numbers, content of text message responses, and documentation of incoming and outgoing messages will be accessible to study staff through a password protected administrative web page available only over an encrypted connection (SSL).

Adverse Event Reporting

HHS definition for "adverse events" will be used for this study (https://www.hhs.gov/ohrp/regulations-and-policy/guidance/reviewing-unanticipated-problems/index.html#AA). Adverse event is defined as any untoward or unfavorable medical occurrence in a participant, including any abnormal sign, symptom, or disease temporally associated with participation in the research. A serious adverse event is defined as any adverse event temporally associated with the subject's participation in research that: 1) results in death; 2) is life-threatening; 3) requires inpatient hospitalization or prolongation of existing hospitalization; 4) results in a persistent or significant disability/incapacity; 5) results in congenital anomaly/birth defect; or 6) any other adverse event that may jeopardize the participant's health and may require medical or surgical intervention to prevent one of the other outcomes listed above. Unanticipated problem includes any incident, experience, or outcomes that is: 1) unexpected given the research procedures and the characteristics of the subject population being studied; 2) related or possibly related to a subject's participation in the research; and 3) suggest that research places subjects or others at a greater risk of harm related to the research than was previously known or recognized.

Adverse events, serious adverse events, and unanticipated problems are unlikely in this sociobehavioral only intervention, but any potential events will be reported immediately upon discovery by study staff to the Principal Investigator who will notify the IRB within 24 hours.

Regulatory and Ethical Considerations

This study targets health topic that is currently receiving significant media and regulatory attention (e-cigarette use). We do not anticipate any regulatory changes that may occur during the study period to impact study conduct as our study is aimed at evaluating a cessation program for a product that may become less available and fits well into the currently developing regulatory environment.

The assessment-only arm was selected as the most ethical control group for the current study that allows for evaluation of the intervention while keeping control arm participants engaged. All control arm participants will have access to the program at the conclusion of the study if they are interested.

Our payment structure was developed to incentivize participants for their time and increase retention while not creating any coercion. Thus the incentives are nominal amounts, but provides choices for participants that we hypothesize will lead to great retention.

The current study is as a low risk, sociobehavioral only intervention and we do not anticipate any other ethical considerations.

<u>RECRUITMENT MATERIALS</u>

Sample Recruitment Advertisement Text Options

- *Do you JUUL? Interested in quitting? You may be eligible for a paid study! Click here for more info.*
- *Do you vape? Thinking about quitting? You may be eligible for a paid study. Click here for more info.*
- *If you've been thinking about quitting JUUL (or whatever vape you used to love), you may be eligible for a paid study. Click to learn more!*

Study Information Page Text

We are evaluating a new quit vaping/JUULing program developed by Truth Initiative, America's largest nonprofit public health organization dedicated to making tobacco use a thing of the past. We need your help to make it the best it can be!

What would I be asked to do?

1. *Answer a few questions today to see if you're eligible*
2. *Complete an 8-10 minute survey today about your experience vaping*
3. *Sign up for a text message program and respond to the first message*
4. *Answer a few text message questions over the next 6 months about your vaping/JUULing*
5. *Complete 2 surveys, 1month and 7 months from now to tell us about your vaping/JUULing*

Why should I join the study?

Your feedback will be invaluable in helping us evaluate our program.

*To thank you, you'll receive $5 each time you tell us about your vaping status via text message, and $30 when you complete the 1-month and 7-month surveys. That's a **total possible $95**.*

This program may also help you in your own efforts to quit vaping/JUULing!

How will my information be used?

Your name and the fact that you were in the study will be kept completely confidential. Results of this research study will be used to improve the program. They will also be published in scientific reports and shared at academic conferences.

Ready to answer a few questions to see if you are eligible?

***** CHECK MY ELIGIBILITY *****

truth initiative
INSPIRING TOBACCO-FREE LIVES

WHO WE ARE ▾ | WHAT WE DO ▾ | OUR TOP ISSUES ▾ | RESEARCH & RESOURCES ▾ | GET INVOLVED ▾ | SEARCH

Home » Get Involved » Participate in Our Research » **Quit Vaping Study**

STUDY

Quit Vaping Study

Nov. 06, **2019**

We are evaluating a new quit vaping/JUULing program developed by Truth Initiative, America's largest nonprofit public health organization dedicated to making tobacco use a thing of the past. We need your help to make it the best it can be!

CHECK MY ELIGIBILITY →

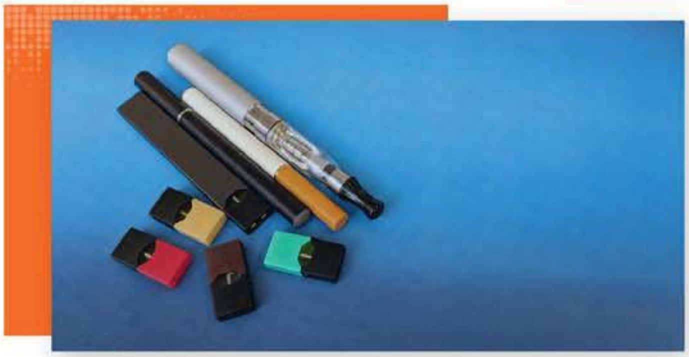

What would I be asked to do?

1. Answer a few questions today to see if you're eligible
2. Complete an 8-10 minute survey today about your experience vaping
3. Sign up for a text message program and respond to the first message
4. Answer a few text message questions over the next 6 months about your vaping/JUULing
5. Complete 2 surveys, 1 month and 7 months from now, to tell us about your vaping/JUULing

Why should I join the study?

Your feedback will be invaluable in helping us evaluate our program.

To thank you, you'll receive $5 each time you tell us about your vaping status via text message, and $30 when you complete the 1-month and 7-month surveys. That's a **total possible $95**.

This program may also help you in your own efforts to quit vaping/JUULing!

How will my information be used?

Your name and the fact that you were in the study will be kept completely confidential. Results of this research study will be used to improve the program. They will also be published in scientific reports and shared at academic conferences.

Ready to answer a few questions to see if you are eligible?

CHECK MY ELIGIBILITY →

RESEARCH AND EVALUATION

Learn more about that Truth Initiative Schroeder Institute®

Sign up for our newsletter

Enter your email address

Sign up

truth initiative
INSPIRING TOBACCO-FREE LIVES

900 G Street, NW
Fourth Floor
Washington, DC 20001
202.454.5555

Follow us

Annual reporting
Careers
Contact us
Website policies
Conflict of interest

ASSESSMENTS

Screening Questionnaire

*** Bolded response options required for eligibility**

Please note that the terms "vape" or "vaping" in this survey refer to use of all vaping products, including JUUL, mods, and other e-cigarettes.

1. **How old are you? (18-24 required for eligibility)**
   ____ [numeric entry]

2. **How often do you vape <u>nicotine</u>?**
   1 = Daily or almost daily
   2 = Less than daily, but at least once a week
   3 = Less than weekly, but at least once a month
   4 = Less than monthly
   5 = Not at all
   6 = Don't know

3. How often do you vape **<u>marijuana, marijuana concentrate, marijuana waxes, THC, CBD or hash oil</u>**?
   1 = Daily or almost daily
   2 = Less than daily, but at least once a week
   3 = Less than weekly, but at least once a month
   4 = Less than monthly
   5 = Not at all
   6 = Don't know

4. **Are you seriously thinking about quitting vaping?**
   1 = Yes, during the next 30 days
   2 = Yes, in the next 6 months
   3 = Yes, in the next 12 months
   4 = Yes, but not in the next 12 months
   5 = No, I'm not thinking about quitting vaping

5. What is the highest level of school/degree you have completed?
   1 = Less than high school (no diploma)
   2 = GED or equivalent
   3 = High school graduate
   4 = Some college or technical school
   5 = College graduate
   6 = Graduate degree
   7 = Prefer not to say

6. Considering your income and income from any people who help you, what is your personal financial situation?
   1 = Live comfortably
   2 = Meet needs with a little left
   3 = Just meet basic expenses
   4 = Don't meet basic expenses

7. What is your gender identity?
   1 = Male
   2 = Female
   3 = Non-binary
   4 = Other
   5 = Prefer not to say

8. Which of the following would you say is your race? Check all that apply.
   1 = Black or African American
   2 = American Indian or Alaska Native
   3 = Asian
   4 = Native Hawaiian/Other Pacific Islander
   5 = White
   6 = Other
   7 = Prefer not to say

9. Are you Hispanic, Latino/a, or Spanish origin?
   0 = No
   1 = Yes
   2 = Prefer not to say

10. Which of the following best describes you?
   1 = Straight
   2 = Gay or lesbian
   3 = Bisexual
   4 = Other
   5 = Prefer not to say

---

*Participants who are **not eligible** (under age 18, no e-cig with nicotine use in past 30 days OR not interested in quitting in the next 30 days) will see the following message.*

Thanks for your interest in the study but unfortunately you are not eligible. For free help quitting vaping, text DITCHJUUL to 88709.

****

*Participants who are **eligible** will see the following message and receive a link via email to complete the Baseline Survey.*

You are eligible for the study! The next step is a short survey. Please enter your email and we'll send a link to the survey.

Email: _________________
Confirm your email: ________________

What is the cell phone number where you want to receive our text messages? ****Please make sure to use this same phone number when signing up to enroll in the study*****

Cell phone number: ____________________

Baseline Assessment

****The terms “vape” or “vaping” in this survey refer to all vaping products, including JUUL, mods, and other e-cigarettes.****

ADDITIONAL DEMOGRAPHIC QUESTIONS

1. Are you currently a student?
   1 = No
   2 = Yes
   3 = Prefer not to say

2. [SKIP PATTERN, Only ask if YES to Q1] What school do you currently attend?

   1 = High school
   2 = Vocational/technical school
   3 = Community college or junior college
   4 = 4-year college or university
   5 = Graduate or professional school
   6 = Other (specify): _____________
   7 = Prefer not to say

3. Which best describes your current job/paid employment status?
   1 = Work full-time (35 hrs/wk or more)
   2 = Work part-time (15-34 hrs/wk)
   3 = Work part-time (less than 15 hrs/wk)
   4 = Don’t currently work for pay
   5 = Prefer not to say

E-CIGARETTE USE HISTORY

These questions are all about vaping NICOTINE.

4. How many days per week do you vape?
   [Integer response, 1-7]

5. On average, how many puffs/hits on your vape do you take on a typical day?
   [Integer response, any]

6. On average, how much do you spend a week on vaping?
   $[integer response, any]

CONFIDENCE/MOTIVATION/SELF-EFFICACY

7. How much do you want to quit vaping?
   1 = Not at all
   10 = Very much

8. How confident are you that you can quit vaping?
   1 = Not at all
   10 = Very much

9. Which of these statements apply to you? Select all that apply.
   1 = I vape to cope with stress
   2 = I am scared about quitting vaping
   3 = Sometimes I think quitting vaping will be impossible
   4 = I want to quit vaping but I don’t know how

PAST QUIT ATTEMPTS

These questions are all about vaping NICOTINE.

10. In the past 12 months, how many times have you stopped vaping for one day or longer?
    1 = None
    2 = 1 time
    3 = 2 times
    4 = 3 to 5 times
    5 = 6 to 9 times
    6 = 10 or more times

11. In the past 12 months, has a doctor/dentist/etc advised you to quit vaping?
    0 = No
    1 = Yes
    2 = I haven't seen a doctor/dentist/etc in the past 12 months

12. How much do you want to quit vaping because…

…It's bad for your health.	1 = Not at all, 10 = Very much
…It's too expensive.	1 = Not at all, 10 = Very much
…It's difficult to get (e.g. device, e-liquid, pods, cartridges)	1 = Not at all, 10 = Very much
…Someone else is making me quit	1 = Not at all, 10 = Very much
…I want to quit for someone else.	1 = Not at all, 10 = Very much
…I don't want to be punished for vaping	1 = Not at all, 10 = Very much
…I don't want to be addicted.	1 = Not at all, 10 = Very much
…It's just wrong for me to use it.	1 = Not at all, 10 = Very much
Other: _______	1 = Not at all, 10 = Very much

E-CIGARETTE DEPENDENCE

	Never	Rarely	Sometimes	Often	Almost always
13. I find myself reaching for my vape without thinking about it.	0	1	2	3	4
14. I drop everything to go out and buy a vape or e-liquid	0	1	2	3	4
15. I vape more before going into a situation where vaping is not allowed.	0	1	2	3	4
16. When I haven't been able to vape for a few hours, the craving gets intolerable.	0	1	2	3	4

17. Have you ever tried to stop vaping, but couldn't?	Yes	No
18. Do you vape now because it is really hard to quit?		
19. Have you ever felt like you were addicted to vaping?		
20. Do you ever have strong cravings to vape?		
21. Have you ever felt like you really needed to vape?		
22. Is it hard to keep from vaping in places where you are not supposed to, like school or work?		
When you tried to stop vaping (or when you haven't vaped in awhile)…		
23. Did you find it hard to concentrate because you couldn't vape?		
24. Did you feel more irritable because you couldn't vape?		
25. Did you feel a strong need or urge to vape?		
26. Did you feel nervous, restless or anxious because you couldn't vape?		

27. How soon as you wake up do you first vape?
    1 = Within 5 minutes
    2 = 6 to 30 minutes
    3 = 31 to 60 minutes
    4 = After 60 minutes

28. How aware are you of media reports on illnesses and deaths related to vaping?
    1 = Not at all
    10 = Very much

29. How worried are you about the consequences of vaping?
    1 = Not at all
    10 = Very much

30. Have you experienced any of the following vaping-related symptoms? Select all that apply.
    1 = Respiratory symptoms (e.g., cough, chest pain, and shortness of breath)
    2 = Gastrointestinal symptoms (e.g., abdominal pain, nausea, vomiting, and diarrhea)
    3 = Fever, chills, or weight loss

<u>SOCIAL SUPPORT</u>

31. Of the 5 closest friends you spend time with on a regular basis, how many of them vape NICOTINE?
    [Dropdown 1-5]

32. Does anyone who lives with you vape NICOTINE?
    1 = Yes
    2 = No

33. Does anyone who lives with you now use other tobacco products, like cigarettes, cigars, chewing tobacco, etc?
    1 = Yes
    2 = No

34. How many of your <u>friends</u> are supportive of your desire to quit vaping?
    1 = All of them
    2 = Most of them
    3 = About half of them
    4 = Very few of them
    5 = None of them
    6 = No one knows I want to qui

35. How many of your <u>family members</u> are supportive of your desire to quit vaping?
    1 = All of them
    2 = Most of them
    3 = About half of them
    4 = Very few of them
    5 = None of them
    6 = No one knows I want to quit

36. Which of the following statements apply to you? Select all that apply.
    1 = I feel pressured to vape from my friends
    2 = I have been teased/bullied for wanting to quit
    3 = Most people my age who vape don't want to quit
    4 = Thinking about quitting makes me feel alone

37. Out of 100 people your age, how many of them do you think vape?
    1 = Nearly all of them
    2 = Most of them

3 = About half of them
4 = Less than half of them
5 = Almost none of them

38. Out of 100 people your age who vape, how many of them do you think want to quit?
1 = Nearly all of them
2 = Most of them
3 = About half of them
4 = Less than half of them
5 = Almost none of them

OTHER SUBSTANCE USE

39. During the past 30-days (including today), did you use any of these products, even once?

	No	Yes
Large cigars (like Cohiba or Romeo y Julieta)	0	1
Little cigars/cigarillos (e.g., Black and Milds, Swisher Sweets, Dutch Masters, Phillies Blunts)	0	1
Cigarettes	0	1
Hookah/shisha/waterpipe (hookah tobacco)	0	1
Chewing tobacco, snuff, or snus	0	1
Marijuana/cannabis	0	1

40. Have you ever, even once, had a drink of any type of alcoholic beverage? Please do not include times when you only had a sip or two from a drink.
1 = Yes
2 = No

41. How long has it been since you last drank an alcoholic beverage?
1 = Within the past 30 days
2 = More than 30 days but within the past 12 months
3 = More than 12 months ago

42. What's your best estimate of the # of days you drank alcohol during the past 30 days?
1 = 1 or 2 days
2 = 3 to 5 days
3 = 6 to 9 days
4 = 10 to 19 days
5 = 20 to 29 days
6 = All 30 days

43. On the days you drank in the past 30 days, how many drinks did you usually have each day?

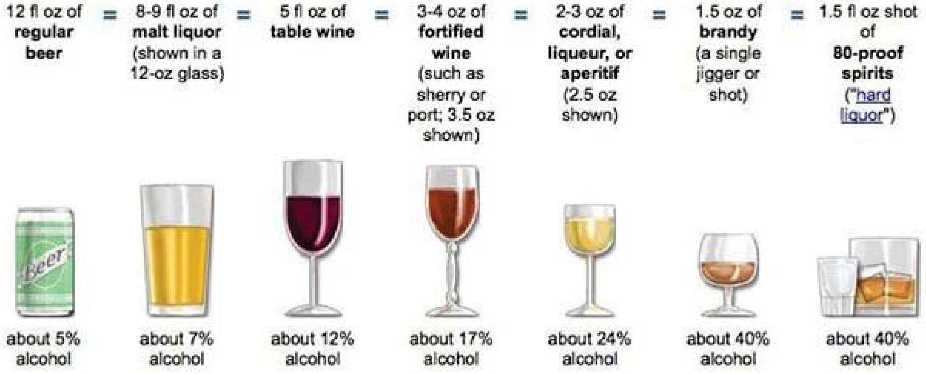

______ drinks

44. In the past 30 days, on how many days did you have 4 (females)/5 (males) or more drinks on the same occasion?
______ days

MENTAL HEALTH CO-MORBIDITIES

45. In the last 2 weeks, how often have you been bothered by any of the following?

	Not at all	Several days	More than half the days	Nearly every day
Little interest or pleasure in doing things	0	1	2	3
Feeling down, depressed, or hopeless	0	1	2	3
Feeling nervous, anxious or on edge	0	1	2	3
Not being able to stop or control worrying	0	1	2	3

46. What's your MAIN reason for joining this research study?
1 = I want to learn more about vaping
2 = I want to quit vaping
3 = I want to make money
4 = I'm interested in research

Follow-Up Assessment
* Only items not repeated from Baseline Assessment are noted below

ABSTINENCE STATUS AND MOTIVATION TO QUIT

OK, let's get started!

1. In the past 30 days, did you vape at all, even a puff of someone else's?
   0 = No
   1 = Yes

2. [Skip logic, only ask if Q1 = Yes] Have you used any vape, even one puff, in the past 7 days?
   0 = No
   1 = Yes

3. [Skip logic, only ask if Q1 or Q2 = No] When was the last time you vaped?
   [Enter date]

QUIT METHODS

4. [Since you joined the study a month ago/In the last 6 months], how many times have you stopped vaping – not even a puff – for one day or longer because you were trying to quit for good?
   ______ [numeric entry]

5. [Skip logic, only ask if response to Q4 > 0]   In the past [1/6 months], which of the following things have you tried to quit vaping? Check all the categories that apply.
   - Nicotine replacement medicine (patch, gum, lozenge, nasal spray, inhaler)
   - Prescription medicine like Zyban/bupropion or Varencline/Chantix
   - Switching to cigarettes
   - Switching to chewing tobacco or snuff

CUSTOMER SATISFACTION QUESTIONS

6. Overall, how satisfied are you with the guidance and information you received from the quit vaping text message program?
   1 = Very satisfied
   2 = Somewhat satisfied
   3 = A little satisfied
   4 = Not at all satisfied

7. How likely would you be to recommend the quit vaping text message program to family or friends who want to quit JUULing/vaping??
   0-10 scale

8. Was the number of text messages from the quit vaping text message program too many, too few, or just right?
   1 = Too few
   2 = Just right
   3 = Too many

9. Based on your experience with the text messages from This is Quitting, how much do you agree/disagree with the statements below?

	Completely agree (4)	Agree somewhat (3)	Disagree somewhat (2)	Completely disagree (1)
They helped me stay on track with quitting				
They helped me feel more confident about quitting				
They were written personally for me				

They suggested quitting strategies that were new to me				
They made me feel less alone in quitting				
They made me feel that I knew the right steps to take to quit				
I liked being able to interact with the text messages				
I liked reading tips from other people who had quit				

OPEN ENDED QUESTION

10. Awesome, you're done! Any other feedback you want to give us? We're always looking to improve, so your ideas are welcome.
    ____________________ [open ended text entry]

REMINDER MESSAGE RE: PAYMENT

Thanks again for your time and feedback. Click the following link to select the incentives that you want to receive. If you completed your survey within 24 hours of receiving the notice, we will send you a separate note to about your extra $10 bonus. If you have any questions, please send us a note at thisisquitting@truthinitiative.org.

Thanks!

~ Sarah (Research Manager for the Truth Initiative Quit Vaping Study)

****LINK TO RYBBON****

SAMPLE TEXT MESSAGES

Message type	Sample messages
No quit date	
Building skills	For one day, keep track of when you [device] and what the situations are that make you crave or [device]. By knowing your triggers, you can make plans to deal with those *without* your [device].
Confidence	Anon says "You are strong, you deserve freedom from addiction. And it's time for us to own that." You CAN do this. Maybe right now isn't the right time for you to quit, but stick with me for a few more weeks -- I'll send tips to help you feel more ready.
Has quit date	
Encourage and support	Benji says "If I can quit anyone can quit there's no way anyone is ever been as addicted as I was." No matter how much you use an [device], it's definitely possible to quit.
Skill- and self-efficacy building exercises	AnnetteMM says "Gather preparation supplies ahead of time. I bought twizzlers and tootsie pops." Tell me what you're going to buy to put in your mouth instead of a [device] next week.
Coping strategies	If you used to [device] a lot, you might feel anxious or sweaty today. Keep taking deep breaths and drinking cold water. It's going to be ok -- you can get through today.
Information about risks of vaping	Pam loves to walk and hike, and her [device] felt like it got her heart racing or she felt nervous. Quitting will help you feel like the best version of you.
Benefits of quitting	Brittany suggests keeping track of how much money you used to spend on your [device] so you can see what you're saving. Makes you feel rich!
Cutting down to quit	SpartanGirl117 says "It takes time to break the habit so be patient. Each time you beat a craving, your brain is relearning a new habit of NOT using your [device]"
On-demand support	
COPE	tjh.upnext says "I like to keep a water on me. End one addiction with another one. Drink water when you are having an urge. Healthy addiction!"
STRESS	Chase says "It's alright take it a few minutes at a time if you are stressed remember it only last a few minutes at a time"
SLIP	Practice makes progress! This is a process, and you're still learning what the right way is for YOU to quit.
MORE<br>• FEELS<br>• TIPS	• jklause says "Remember why your quitting and don't look back. Keep going strong and you'll be able to achieve sobriety."<br>• Anonymous says "Make a list of reasons as to why you want to quit. Have it on your phone or carry it around, and read over it during a craving."

PARTICIPANT COMMUNICATION

Confirmation Email with Link to Baseline

Subject: We want you in the Truth Initiative Quit Vaping Study!

Dear {{ participant.name }},

We want you to participate in the Truth Initiative Quit Vaping Study! Your participation will help us build the best quit vaping program possible.

Please fill out the baseline survey by clicking on the link below. **You must complete the survey within 24 hours to fully enroll in the study!**

{{ survey_link }}

On behalf of the Truth Initiative Quit Vaping Study team, thank you!

Sarah Cha
Project Manager | Truth Initiative Quit Vaping Study | (202) 525-6473

P.S. If for any reason your survey session expires before completing the survey, your answers will be saved and you can finish the survey by clicking on this link again.

Survey Invitations

Email 1

Subject: It's Sarah from the Truth Initiative Quit Vaping Study – Your survey is waiting!

Dear {{ name }},

It's Sarah from the Truth Initiative Quit Vaping Study - thanks for being a part of our research! It's time for your follow-up survey. It will only take around 10 minutes.

Click or copy/paste the following link to access your unique survey: **{{ followup_url }}**

To thank you for your time, we will email you a $20 gift code to use at an online retailer of your choice. BONUS! We'll add an extra $10 bonus if you complete the survey within the next 24 hours!

We're evaluating this program, **not you**. So if you're still vaping or haven't totally quit yet, we still value your response.

Sincerely,

Sarah Cha
Project Manager | Truth Initiative Quit Vaping Study | (202) 525-6473

Email 2

Subject: Your response matters! Take the Truth Initiative Quit Vaping Study survey

Dear {{ name }},

Just a quick reminder to complete the follow-up survey for the Truth Initiative Quit Vaping Study. It takes less than 10 minutes to complete and I'll send you a $20 online gift card (think Amazon, Apple, Target, Starbucks… your choice!).

Click or copy/paste the following link to access your unique survey: **{{ followup_url }}**

Thanks so much,

Sarah Cha
Project Manager | Truth Initiative Quit Vaping Study | (202) 525-6473

P.S. Even if you haven't quit vaping, that's OK! Your responses still really matter to our study.

Email 3

Subject: $20 to complete the Truth Initiative Quit Vaping Survey

Dear {{ name }},

I promise: no more emails after this. Please take 10 minutes to fill out the follow-up survey for the Truth Initiative Quit Vaping Study. I'll send you a $20 gift card to the retailer of your choice.

Click or copy/paste the following link to access your unique survey: **{{ followup_url }}**

Your responses are truly valuable to our research, that will help thousands of other young vapers quit. Thank you!

Sarah Cha
Project Manager | Truth Initiative Quit Vaping Study | (202) 525-6473

P.S. Not sure if you should take the survey because you haven't quit or are vaping sometimes? Easy answer: YES! Your responses mean so much to our research.

Text Message Assessments

Time	Original message	Response to anyone sending a response
**2 weeks**	Checking in: Have you cut down how much you vape nicotine in the past 2 weeks? Respond w/letter: A=I still use the same amount, B=I use less, C=I don't use at all anymore	Thanks for your time in responding, it's really helpful for our study! We'll be sending you a link to redeem your $5 incentive shortly.
**1 month – 6 months**	How's the quit going? When was the last time you vaped nicotine, even a puff of someone else's? Respond w/ letter: A- in the past 7 days, B- 8-30 days ago, C- More than 30 days ago	Thanks for your time in responding, it's really helpful for our study! We'll be sending you a link to redeem your $5 incentive shortly.

REFERENCES

1. Surgeon General's Advisory on E-cigarette Use Among Youth. 2018. at https://e-cigarettes.surgeongeneral.gov/documents/surgeon-generals-advisory-on-e-cigarette-use-among-youth-2018.pdf.)
2. Centers for Disease Control and Prevention. Electronic cigarettes: What's the bottom line? 2019.
3. Vallone DM, Bennett M, Xiao H, Pitzer L, Hair EC. Prevalence and correlates of JUUL use among a national sample of youth and young adults. Tobacco control 2018.
4. In: Eaton DL, Kwan LY, Stratton K, eds. Public Health Consequences of E-Cigarettes. Washington (DC)2018.
5. Primack BA, Shensa A, Sidani JE, et al. Initiation of Traditional Cigarette Smoking after Electronic Cigarette Use Among Tobacco-Naive US Young Adults. Am J Med 2018;131:443 e1- e9.
6. Gray KM, Baker NL, McClure EA, et al. Efficacy and Safety of Varenicline for Adolescent Smoking Cessation: A Randomized Clinical Trial. JAMA Pediatr 2019.
7. Fanshawe TR, Halliwell W, Lindson N, Aveyard P, Livingstone-Banks J, Hartmann-Boyce J. Tobacco cessation interventions for young people. Cochrane Database Syst Rev 2017;11:CD003289.
8. Whittaker R, McRobbie H, Bullen C, Rodgers A, Gu Y, Dobson R. Mobile phone text messaging and app-based interventions for smoking cessation. Cochrane Database Syst Rev 2019;10:CD006611.
9. Graham AL, Jacobs MA, Amato MS. Engagement and 3-month outcomes from a digital e-cigarette cessation program in a cohort of 27,000 teens and young adults. Nicotine & tobacco research : official journal of the Society for Research on Nicotine and Tobacco 2019.
10. Mendel J. Finding truth in smoking status self-reporting: A formative evaluation of reporting modality used in the Be Free Smoking Study of the Text2Quit program: School of Public Health and Health Services, The George Washington University; 2013.
11. Graham AL, Papandonatos GD. Reliability of internet- versus telephone-administered questionnaires in a diverse sample of smokers. Journal of medical Internet research 2008;10:e8.
12. Graham AL, Papandonatos GD, Bock BC, et al. Internet- vs. telephone-administered questionnaires in a randomized trial of smoking cessation. Nicotine & tobacco research : official journal of the Society for Research on Nicotine and Tobacco 2006;8 Suppl 1:S49-57.
13. Pearson JL, Hitchman SC, Brose LS, et al. Recommended core items to assess e-cigarette use in population-based surveys. Tobacco control 2018;27:341-6.
14. Bold KW, Sussman S, O'Malley SS, et al. Measuring E-cigarette dependence: Initial guidance. Addictive behaviors 2018;79:213-8.
15. Centers for Disease Control and Prevention. National Youth Tobacco Survey Questionnaire. 2018.
16. Morean M, Krishnan-Sarin S, O'Malley SS. Comparing cigarette and e-cigarette dependence and predicting frequency of smoking and e-cigarette use in dual-users of cigarettes and e-cigarettes. Addictive behaviors 2018;87:92-6.
17. Case KR, Mantey DS, Creamer MR, Harrell MB, Kelder SH, Perry CL. E-cigarette- specific symptoms of nicotine dependence among Texas adolescents. Addictive behaviors 2018;84:57-61.
18. Substance Abuse and Mental Health Services Administration. National Survey on Drug Use and Health Questionnaire. 2018.
19. National Health Interview Survey. 2015. (Accessed 06/23/2015, 2015, at http://www.cdc.gov/nchs/nhis/nhis_questionnaires.htm.)
20. Abroms LC, Boal AL, Simmens SJ, Mendel JA, Windsor RA. A Randomized Trial of Text2Quit: A Text Messaging Program for Smoking Cessation. American journal of preventive medicine 2014.
21. Strecher VJ, McClure J, Alexander G, et al. The role of engagement in a tailored web-based smoking cessation program: randomized controlled trial. Journal of medical Internet research 2008;10:e36.
22. Dijkstra A, Ballast K. Personalization and perceived personal relevance in computer-tailored persuasion in smoking cessation. British journal of health psychology 2012;17:60-73.
23. Blankers M, Smit ES, van der Pol P, de Vries H, Hoving C, van Laar M. The Missing=Smoking Assumption: A Fallacy in Internet-Based Smoking Cessation Trials? Nicotine & tobacco research : official journal of the Society for Research on Nicotine and Tobacco 2016;18:25-33.

APPENDIX A

Privacy and Terms of Service

HERE COMES A LOT OF **LEGAL MUMBO JUMBO**. SO, EVEN IF YOU'RE BORED TO TEARS, READ IT AND THEN CHECK OUT THE REST OF OUR WEBSITE.

Privacy Policy

www.thetruth.com is a website of Truth Initiative, which is dedicated to achieving a culture where all youth and young adults reject tobacco. We respect your privacy and have created this Privacy Policy to explain what information we collect through the website and how we use it. If you have any questions about this policy, please contact us.

Non-Personally Identifiable User Information

We automatically collect non-personally identifiable information about our site's users, including the domain name and IP address of visitors, the browser and operating system used, the date and time of each visit and the source of referral. This information does not identify individual visitors, and it is used for our own research purposes and to improve the site's content, structure, and security.

Cookies

Cookies are information files that allow websites to remember users' activities on a website. When you visit our site, we may send one or more cookies to your computer that uniquely identifies your browser. We use the information we learn from cookies to improve the quality of our service, for example, by tracking user trends. We do not use cookies to track user activity on an individual basis. Most browsers are initially set up to accept cookies, but you can reset your browser to refuse all cookies or to indicate when a cookie is being sent. However, some of our features and services may not function properly if your cookies are disabled.

Personally Identifiable Information

We collect personally identifiable information from you through the site (such as your name, mailing address or email address) when you choose to sign up for our newsletter or otherwise voluntarily provide such information to us. We use your personally identifiable information to create a customized digital experience through the various truth platforms, for analytics, personalization and tracking, and for the purpose for which it was provided, (for example, to add you to our newsletter distribution list, to respond to a query or to fulfill a purchase of truth® merch). If you provide us with your email address, we may begin sending you emails with information that we think will be of interest to you. If, at any time, you wish to stop receiving emails from us, please follow the unsubscribe instructions at the bottom of any email you receive. If you provide your cell phone number to us, you consent to receive autodialed messages and texts at that number on any subject from us, unless and until you request that such messages stop.

We also use personal information about users to our website to better understand how people use our site so that we can improve the website and its offerings.

Unfortunately, given the complexity of privacy regulations outside the United States, we do not profile, personalize, track or create customized digital experience for IP addresses that appear to come from outside the United States. As a result, you may not have the benefit of full digital platform experience.

Security

We take appropriate security measures to protect against unauthorized access to or disclosure or destruction of our users' data. We restrict access to personal information to our employees, contractors, and agents who need to know that information in order to perform their jobs. Nonetheless, we cannot guarantee that your information is 100% secure.

Sharing Personal Information

We may share your personal information with other like-minded organizations, government officials or other policy-makers, or the press if we believe it will help advance our mission of achieving a culture where all youth and young adults reject tobacco and drugs.

We may also share your information if you provide the information in a context where you would expect the information to be shared. For instance, if you choose to sign a truth-sponsored petition with your name and email address, we may share that information with the party receiving the petition. We may use third party service providers to help us operate our website or administer activities on our behalf. Examples may include fulfilling orders, delivering packages, removing repetitive information from customer lists, processing credit card payments and providing customer service. We may share personal information about our users with these third parties for those limited purposes, but we require that such third parties protect the confidentiality of user information and use it for no other purpose.

In addition, we will share personally identifiable information about our users when required to do so by law, or in the good faith belief that such action is necessary to comply with state and federal laws or to respond to a court order, subpoena, or search warrant. We will also share personally identifiable information if we believe it is necessary to protect the rights, property and safety of us or others.

We may also share personally identifiable information in connection with, or during negotiations of, a merger, sale of company assets, financing or acquisition of all or a portion of our business to another entity. Moreover, we may share personally identifiable information about a user upon the user's consent.

We may share generic aggregated demographic information not linked to any personally identifiable information regarding our website users with third parties.

Third Party Websites

We may reference mobile applications ("apps") or provide links from our website to other websites. We do not control the content or links that appear on these third party websites/apps, and we are not responsible for the practices employed by websites linked to or references to apps from our site. For instance, these other websites/apps may use cookies or other methods to track and profile people. These third-party websites and services have their own privacy policies. Browsing and interaction on any other website and/or app, including websites that have a link to or apps referenced from our site, is subject to that website's/app's own terms and policies. **This Privacy Policy covers only information collected through the use of this website**. In addition, we do not currently respond to web browser "do not track" signals.

Adobe Cloud Marketing Coop

Truth Initiative participates in the Adobe Marketing Cloud Device Co-op to better understand how you use our website and apps across the various devices you use, and to deliver tailored promotions and communications. Learn more at https://cross-device-privacy.adobe.com about how Adobe does this.

Compliance with Children's Online Privacy Protection Act

Protecting the privacy of the young is especially important. For that reason, we never collect information at the website from those we know are under 13, and no part of the website is structured to attract anyone under 13. If we become aware of having information about anyone under 13, we will promptly delete the information, unless we obtain consent from the parent or legal guardian of the child to retain such information.

SPECIAL PROVISIONS FOR THIS IS QUITTING PROGRAM

We are excited that you have decided to use This is Quitting to achieve your vaping cessation goals. We are constantly trying to improve our web and mobile tools to help people quit vaping. To further that goal, we collect data about your use of This is Quitting services (e.g., number and frequency of program use, the content of your texts, etc.).

Data collected from the program may be used for public health and research purposes to help us build better tools for quitting smoking or vaping.

When you sign up for This is Quitting, you are agreeing that at a later date, we may send you communications and content about other Truth Initiative programs, initiatives, campaigns and messaging. You may unsubscribe to these communications at any time.

We may share with other third-party research partners or sponsoring organizations certain aggregated, de-identified data collected from users without their consent. Such information will not include any personally identifiable information such as your name or phone number.

Information provided as part of This is Quitting does not constitute medical advice. This is Quitting services are not to be used to make diagnoses, prescribe medicine or provide treatment, and should not be relied upon as a substitute for consultations with qualified health professionals who are familiar with your individual medical needs. The This is Quitting program is provided as a self-help service to provide you with support while quitting vaping. Please contact your health care provider with any concerns about your physical or mental health or about whether this program is or is not working for you. We do not promise or guarantee any outcome, including that you will successfully quit vaping.

For versions of This is Quitting offered through a school, healthcare system, public health organization or other sponsoring entity (each, a "Sponsoring Entity"), we may share aggregated, de-identified data about your use of This is Quitting with such Sponsoring Entity.

CHANGES TO THIS PRIVACY POLICY

We may update this Privacy Policy at any time. When we do, we will revise the updated date at the bottom of this page. We encourage our website users to frequently check this page for any changes to stay informed about what information we collect and how we use that information. You acknowledge and agree that it is your responsibility to review this Privacy Policy periodically and become aware of any modifications since your last visit. Your further use of this site following any such modifications constitutes your agreement to the terms of the amended Privacy Policy.

Your Acceptance of These Terms

By using this website, you signify your acceptance of this Privacy Policy. If you do not agree to this policy, please do not use the website. Your continued use of the site following the posting of changes to this Privacy Policy will be deemed your acceptance of those changes.

Terms of Service

Truth Initiative provides the website www.thetruth.com (the "Site") under the following Terms of Service ("Terms"), which may be amended from time to time at our sole discretion. Your use of the Site constitutes your agreement to these Terms.

Intellectual property

Unless otherwise indicated, Truth Initiative owns all legal right, title and interest in and to the Site, including any and all intellectual property rights. All such intellectual property, including without limitation all trademarks of Truth Initiative, may not be used without Truth Initiative's written consent.

User-Provided Content

You, not Truth Initiative, are legally responsible for any material you post on the Site ("Your Content"). You agree to post only content to which you own the copyright (or other applicable rights) or have written permission from the rights owner to post. Your Content also encompasses your name, likeness and/or image, should you post such items to the Site. You agree not to post any material that:

- violates or infringes upon the intellectual property, privacy, publicity or similar rights of others;
- is abusive, offensive, discriminatory, profane, vulgar, obscene, disparaging, defamatory, libelous, slanderous, or contains information known by you to be false, inaccurate, or misleading;
- violates any law, statute, ordinance or regulation or any third party agreement to which you are subject (e.g., Facebook, Twitter, Instagram, Vine, YouTube, Pinterest, etc.);
- contains (i) third party websites; and/or (ii) addresses, email addresses, contact information, phone numbers or other personal identifiable information (other than your own);
- is in the nature of advertising or marketing; and/or
- contains any computer viruses, worms, harmful code, or other potentially damaging computer programs, applications or files.

In addition, by posting Your Content on the Site, you grant Truth Initiative a non-exclusive, perpetual and royalty-free right and license to use, store, display, reproduce, modify, create derivative works, perform, and distribute Your Content in any manner consistent with Truth Initiative's charitable purpose. This means that Your Content will not be treated as confidential and proprietary, and we'll be able to freely use Your Content without attribution or compensation or payment of any kind to you.

Truth Initiative does not endorse or take responsibility for user-provided content. While Truth Initiative does not routinely review user-proved content, Truth Initiative reserves the right, but is not obligated, to remove any information or materials posted on the Site for any or no reason, at its sole discretion.

You Represent and Warrant That:

- You are at least thirteen (13) years of age;
- You own or otherwise lawfully possess all rights in and to Your Content; and
- Your Content does not and will not violate these Terms or cause injury to any person or entity.

Indemnification and Hold Harmless

You will indemnify and hold harmless Truth Initiative and its officers, directors, employees, and agents from all claims arising out of or related to: (i) Your Content; and/or (ii) your use of, or your inability to use, the Site or the information contained therein or any other website(s) to which the Site is linked.

Modifications

Truth Initiative reserves the right to change or terminate any aspect of the Site, including the Site itself and individual user accounts for any or no reason. Truth Initiative also reserves the right to impose limits on certain features of the Site or restrict access to all or parts of the Site without notice or penalty. Additionally, Truth Initiative may revise these Terms from time to time, and the most current version will always be posted on the Site.

Limitation of Liability

TO THE MAXIMUM EXTENT PERMITTED BY LAW, TRUTH INITIATIVE SHALL NOT BE LIABLE FOR ANY INDIRECT, INCIDENTAL, SPECIAL, CONSEQUENTIAL OR PUNITIVE DAMAGES, OR ANY LOSS OF PROFITS OR REVENUES, WHETHER INCURRED DIRECTLY OR INDIRECTLY, OR ANY LOSS OF DATA, USE, GOOD-WILL, OR OTHER INTANGIBLE LOSSES, RESULTING FROM (A) YOUR ACCESS TO OR USE OF OR INABILITY TO ACCESS OR USE THE SITE; (B) ANY CONDUCT OR CONTENT OF ANY THIRD PARTY ON THE SITE, INCLUDING WITHOUT LIMITATION, ANY DEFAMATORY, OFFENSIVE OR ILLEGAL CONDUCT OF OTHER USERS OR THIRD PARTIES; OR (C) UNAUTHORIZED ACCESS, USE OR ALTERATION OF YOUR TRANSMISSIONS OR CONTENT. IN NO EVENT SHALL TRUTH INITIATIVE'S AGGREGATE LIABILITY FOR ALL CLAIMS RELATING TO THE SITE EXCEED ONE HUNDRED U.S. DOLLARS (U.S. $100.00).

Arbitration

For any dispute you have with Truth Initiative regarding the Site shall be resolved by arbitration in accordance with the Commercial Arbitration Rules of the American Arbitration Association and judgment upon the arbitrator's award may be entered in any court having jurisdiction. The prevailing party in any such arbitration shall be entitled to recover reasonable attorney's fees from the other party. However, nothing shall prevent either you or Truth Initiative from seeking injunctive or other equitable relief from the courts for matters related to data security, intellectual property or unauthorized access to the Site. ANY AND ALL CLAIMS MUST BE BROUGHT IN THE PARTIES' INDIVIDUAL CAPACITY, AND NOT AS A PLAINTIFF OR CLASS MEMBER IN ANY PURPORTED CLASS OR REPRESENTATIVE PROCEEDING, AND, UNLESS BOTH PARTIES AGREE OTHERWISE, THE ARBITRATOR MAY NOT CONSOLIDATE MORE THAN ONE PERSON'S CLAIMS. YOU AGREE THAT, BY ENTERING INTO THESE TERMS, YOU AND TRUTH INITIATIVE ARE EACH WAIVING THE RIGHT TO A TRIAL BY JURY OR TO PARTICIPATE IN A CLASS ACTION.

Governing Law and Jurisdiction

The Site is controlled and operated from the United States. These Terms shall be governed by the laws of the District of Columbia, without respect to its conflict of laws principles. You agree to submit to the personal jurisdiction of a local court located in the District of Columbia or the United States District Court for the District of Columbia, for any actions not subject to the arbitration provisions set forth above.

Indemnification and Hold Harmless

You will indemnify and hold harmless Truth Initiative and its officers, directors, employees, and agents from any and all claims arising out of or related to (1) your Content and/or (2) your use of, or your inability to use, this Application or the information contained therein.

No Waiver

Truth Initiative's failure to assert any right or provision under these Terms shall not constitute a waiver of such right or provision.

Contacting Us

If you have any questions about this Privacy Policy, Terms of Service or anything relating to the website, please contact us.

Appendix F. Qualtrics Security Processes

Qualtrics is dedicated to protecting all customer data using industry best standards.
Many of our biggest customers demand the highest levels of data security, and have tested our services to verify that it meets their standards. In each case, we have surpassed expectations and received high praise from large international organizations.

Qualtrics' most important concern is the protection and reliability of customer data. Our servers are protected by high-end firewall systems, and scans are performed regularly to ensure that any vulnerabilities are quickly found and patched. Complete penetration tests are performed yearly. All services have quick failover points and redundant hardware, with complete backups performed nightly.

Most important is our confidential system component design. It uses multiple checks to certify that packets from one subsystem can only be received by a designated subsystem. Access to systems is severely restricted to specific individuals, whose access is monitored and audited for compliance.

Customer data are stored in a specific location; it does not float around in the "cloud." In addition, all data are processed in that location, and are not moved to another jurisdictional area. In other words, if data are collected in the U.S., all data are processed in the U.S.

Qualtrics uses Transport Layer Security (TLS) encryption (also known as HTTPS) for all transmitted data. We can also protect surveys with passwords and HTTP referrer checking. Our services are hosted by trusted data centers that are independently audited using the industry standard SSAE-16 method.

Qualtrics deploys the general requirements set forth by many Federal Acts, including the FISMA Act of 2002. We meet or exceed the minimum requirements as outlined in FIPS Publication 200.

Since our subscribers control their users and their data, it is important for the users to practice sound security practices by using strong account passwords and restricting access to their accounts to authorized persons

Regarding HIPAA, HITECH, and specific data types: Qualtrics provides general research software and other services where all data are processed equally, without regard to how a customer might classify their data. As such, Qualtrics cannot declare or represent any data entered into its services. Any processing of specific data types are purely incidental, and not required to use the services.

HITECH (Health Information Technology for Economic and Clinical Health Act) updated HIPAA rules to ensure that data are properly protected and best security practices followed. Qualtrics safeguards all customer data, and uses secure data centers to ensure the highest protection as per HITECH requirements.

Questions regarding this statement may be sent to Qualtrics Support.

More information can be found at: https://www.qualtrics.com/security-statement/

Qualtrics

Security White Paper Lite

Information Security

A brief overview of privacy, compliance, and operational policies and procedures

February 6, 2017

Version 5.01

© 2016 Qualtrics, LLC

security@qualtrics.com

Before reading this material you must agree to the terms. If you do not agree, then you must destroy or permanently delete this document. This document may not be uploaded to any web site that is accessible to the general public or indexed by public search engines.

Terms & Conditions

This document contains information regarding Qualtrics security posture. It supersedes all previous versions. The Qualtrics security team has created this document to the best of its ability, and does not warrant that it is error-free.

Certain details may have been purposely minimized to protect our intellectual property (IP) or trade secrets.

Although this document is copyrighted, you may distribute this document for the purposes of evaluating Qualtrics' security posture. The full version of this document requires a confidentiality agreement.

Table of Contents

Executive Summary 4
Introduction 6
General Privacy Policies 9
Certifications / Standards 11
Human Resource Policies 13
Network Design, Locations, and Assessments 15
Preventing Unauthorized Access 18
Development Practices 19
Disaster Recovery 21
Business Continuity 22
Backups – Data Retention 23
Incident Response 24
Risk Management 25
Privacy Appendix 26

Executive Summary

If you read nothing else…

This white paper is intended to give the reader an overview of Qualtrics security-related processes and procedures. It describes key security-related processes performed in all areas of the company, and addresses the security measures we've taken to protect each of those processes (such as secure data collection and disaster recovery).

All Data are owned and controlled by Customers. Qualtrics is only a data processor, not a data controller. All products are self-service ("do-it-yourself") whereby the Customer's Users solely determine: 1) what data to collect; 2) from who; 3) from where; 4) for what purpose; and 5) when to delete it. Qualtrics doesn't transfer Data; all Data are stored and processed in a single data center, and only for the purpose of providing the Services.

Qualtrics will only process Data to the extent necessary to provide the Services, and does not disclose any Data to third parties.

Qualtrics treats all Data as highly confidential, and does not classify or represent the Data. In other words, we don't know what information are being collected, and Customers are free to use the software as they wish. We use industry best practices to keep all Data safe from criminals and hackers, and have devised proprietary methods to prevent disclosing Data to the wrong requester due to programming errors.

This document is not static; it will be periodically updated as our processes, procedures, or the security/privacy landscape change.

DEFINITIONS

Throughout this document, "**Data**" means information entered by a survey respondent, User (survey creator), or the information generated by Customers within the Qualtrics platform. A "**Brand Administrator**" is the account manager of the Customer account. An account is also called a "**Brand.**" A "**User**" is a Brand end-user with a Qualtrics login to create, report on, and send surveys, or otherwise utilize the software (a Brand Administrator is also a User). A "**Respondent**" is an individual who responds to surveys created by a User. "**Responses**" are Data collected from surveys taken in web browsers on computer or mobile platforms, or via SMS. A "**Customer**" means an organization that has a business relationship with Qualtrics. "**Services**" refers to the range of services provided by Qualtrics, including the software, email, support, and online resources.

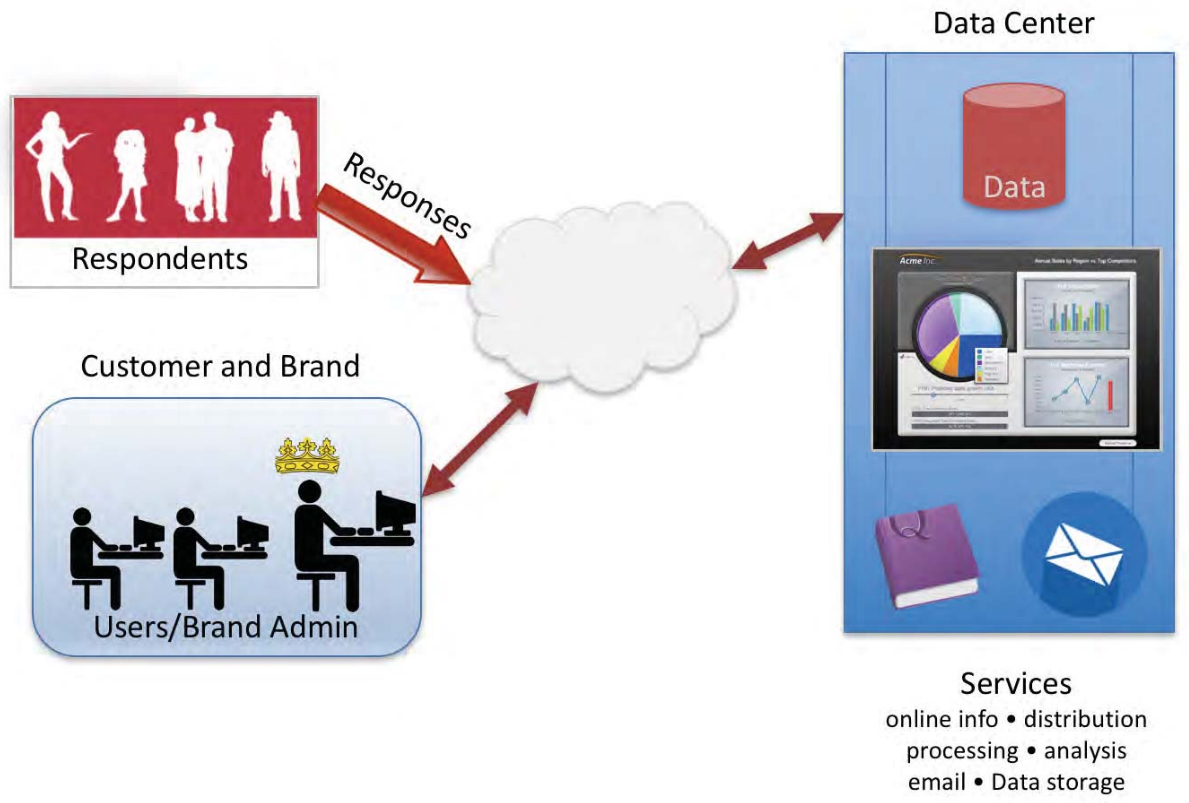

Introduction

WHAT IS QUALTRICS?

Qualtrics is an Application Service Provider (ASP) who provides a platform for creating and distributing online surveys, performing employee evaluations, web site intercepts, and other research services. Software-as-a-Service (SaaS) includes ASP software. The platform records response data, performs analysis, and produces reports on the data. All services are online and require no downloadable software, such as browser plug-ins, JVM, or other components. Only modern JavaScript-enabled internet browsers and an internet connection are required. Qualtrics offers multiple products for online data collection: Research Suite, Vocalize, Employee Engagement/360, and Site Intercept. Services include providing the products and technical support. Surveys are usually taken online within a web browser, with optional SMS surveys and offline methods available for smartphones/tablets.

OVERVIEW OF OUR DATA SECURITY

Qualtrics' most important concerns are the protection and reliability of Data. Our servers are protected by high-end firewall systems, and vulnerability scans are performed regularly. All services have quick failover points with redundant hardware, and complete encrypted backups are performed nightly.

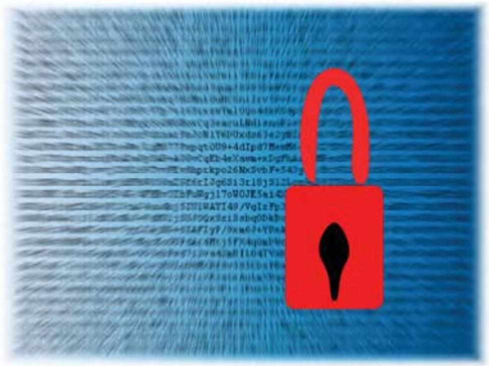

Qualtrics uses Transport Layer Security (TLS) encryption for all transmitted Internet data. Customers may opt to password-protect their surveys, or have unique ID links that are difficult to guess. Our services are hosted by trusted third party data centers that are audited using the industry standard SSAE-16 SOC 1 Type 2 method. All data at rest are protected using sophisticated electronic controls, and data on deprecated hard drives are destroyed by U.S. DOD methods and delivered to a third-party data destruction service.

Qualtrics security practices and operating environments are continuously monitored and assessed. Our processes and procedures to safeguard Data meet or exceed industry standards. The Qualtrics Security and Privacy Officer is accredited by ISC^2 and IAPP (CIPP/US).

Security within the Qualtrics Services

All Qualtrics products are self-service, and enable Customers to control individual permissions of their accounts and surveys. The Customer designates at least one Brand Administrator who manages the Brand, and that person(s) decides who will create, distribute, and analyze the brand's surveys. There is also an option to require surveys to first be approved prior to distribution. Account password strength is also controlled by Brand Administrators.

Our service level standards

Qualtrics serves thousands of worldwide businesses, universities, and other organizations. As a result, Qualtrics must maintain the highest service levels and create environments to minimize downtime. Since 2010, Qualtrics has maintained average up-time of 99.97%.

Disaster recovery plan

Within North America, Qualtrics maintains production servers in geographically and geologically distinct areas. Qualtrics is prepared to quickly shift to unaffected servers in the event of any local catastrophe. In other parts of the world, each data center has a redundant setup nearby, and is maintained by data center staff. More about

disaster recovery and data center locations below.

Our commitment to data security

Keeping Data secure is of paramount importance. Many of our Customers demand the highest levels of data security, and have tested our systems to ensure it meets their standards. In each case, we have surpassed expectations, and received high praise from top companies. All Qualtrics accounts are password protected, and all Data are replicated in real-time. Passwords are salted, then hashed and stored, making them unknown to any Qualtrics employee. Qualtrics IDs may be linked to the Customer's single sign-on services for better User control.

WHO OWNS THE DATA IN QUALTRICS SERVICES?

Customers own and control all Data entered in or collected by Qualtrics Services. This includes survey definitions, response data, panel data, uploaded content such as graphics, user information, and report results/analysis from such data. Qualtrics may collect anonymous usage statistics (such as number of responses collected) for analyzing performance and calculating account quotas.

Qualtrics only processes Data to provide the Services. No Data are ever shared or distributed with a third party except as allowed by the Customer. (Exceptions noted below in Disclosing Customer Information section.) And since Qualtrics products are self-service, Data are essentially invisible to our staff; customers operate on their own accord.

DATA CLASSIFICATION/REPRESENTATION

Qualtrics processes all Data the same using industry best security measures designed to prevent unauthorized access and disclosure. Qualtrics does not represent or attest to Data entered into its Services since all Data and User accounts are controlled by the Customer. Qualtrics does not know what Data are being collected, and therefore cannot classify or treat any Data differently. This is beneficial to our Customers, who solely determine how Data should be interpreted and analyzed.

ASSESSMENTS

Automated vulnerability scans are performed regularly with a commercial security provider. Complete penetration tests are performed yearly by an independent security firm.

CUSTOMER TESTING

With prior consent and signed agreement, Customers have the right to perform non-intrusive vulnerability scans or penetration tests on the Qualtrics software. These tests require coordination between the Customer's account executive, the Customer's IT/security team, and the Qualtrics InfoSec team. All testing must be performed on the staging environment, not the production environment.

SURVEY SECURITY

There are many ways to protect surveys from being "stuffed," or being taking by the wrong respondent. Full details are available on the Qualtrics support web site. Surveys may be sent to specific individuals, require a password, or be taken only by Customer employees. It's up to the Users to determine who should take the survey and what content should be collected. Survey links may be posted on a web page, sent in email, or printed on paper and delivered via certified mail.

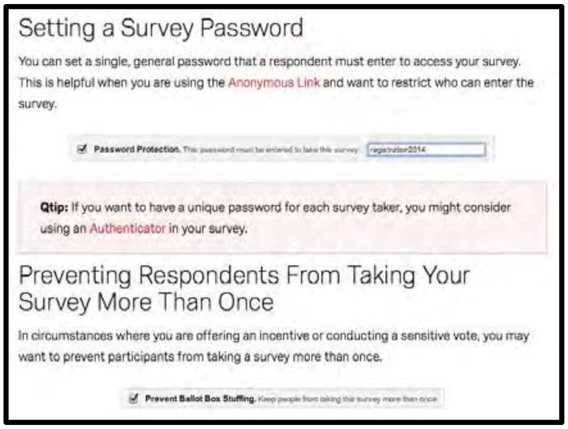

General Privacy Policies

The Qualtrics online privacy policy covers the use and disclosure of personal information that may be collected anytime a user interacts with Qualtrics. Such interactions include visiting any of our web sites, using the Service, or when calling our sales and support departments. A detailed privacy statement is found at the www.qualtrics.com site. In addition, the Terms of Use state acceptable policies regarding the Qualtrics Services.

DISCLOSING CUSTOMER INFORMATION

Qualtrics does not sell or rent Customer or other contact information to other marketers or vendors. Any disclosure of information *within* Qualtrics (as a company) is strictly to assist with technical or service issues. We have high security measures to keep Data safe. We maintain strict control over access to Data. The only employees who are permitted to access Data are those with a business need to know. Qualtrics reserves the right to transfer Customer information within the company throughout the licensing process: for example, from sales staff to accounting.

Qualtrics will only disclose customer information as legally required—either upon a valid court order under applicable law, or to law enforcement or governmental agencies for issues of public importance (for example, national security and time is of the essence). In any case, Customers will be notified as soon as reasonably possible, where allowable by law, if any request is made to disclose their name or any detail of their association with Qualtrics.

HOW WE PROTECT YOUR INFORMATION

Qualtrics takes preventative measures to protect all Customer information, both programmatically and through employee training. All employees must attend yearly security awareness programs (covering privacy, security, and other policies) and sign confidentiality agreements. Security updates and reminders are sent to all employees as required. New employees are also given security training.

RESPONDING TO DATA BREACHES

Privacy-related inquiries are handled by various Qualtrics teams or by calling Qualtrics Support. Qualtrics will assign a case manager and provide all necessary documentation for review. Within a reasonable time, or as stipulated by contractual agreement, the case manager will conduct a formal review, and provide the findings to the User that requested the review. Urgent events are processed as soon as possible, and steps taken to mitigate non-compliance or respond to official inquiries. Conditions set forth in the Qualtrics privacy statement, or any specific agreement between Customer and Qualtrics, will govern specific actions. More details in relevant sections below.

STATUTORY BODY FOR PRIVACY QUESTIONS OR DISPUTES

The Federal Trade Commission has jurisdiction to hear any claims against the organization regarding possible unfair or deceptive practices and violations of laws or regulations governing privacy.

SUBCONTRACTORS

Qualtrics does not use subcontractors to provide its Services.

POLICIES ON THE WEB — ACCEPTABLE USE, PRIVACY, SECURITY

Links to Qualtrics' privacy and security statements, and acceptable use policy are at the bottom of nearly every Qualtrics web page. Most language is based on industry standards, and tweaked to our ASP business model. The Terms of Service must be acknowledged by every User, and uses common language to explain acceptable use of our Service. Any conflicting sections in a Customer signed service agreement supersede the Terms of Service. Qualtrics reserves the right to disable any User account suspected of violating acceptable use terms. This includes uploading

harmful or hateful content (except for valid research purposes), using the Services to "phish" or "spam," or violating the Terms of Service or terms of an executed service agreement between Qualtrics and Customer.

While Qualtrics cannot prevent Customers from entering any specific type of information, prudence and common sense apply. Research software should not be used to store highly sensitive information, such as bank accounts, credit card numbers, social security numbers, criminal records, or genetic information.

> Depending on applicable laws or regulations, it may be necessary for the Customer to de-identify or anonymize sensitive information prior to distributing surveys. Also, it is usually required to receive permission before sensitive information is collected. It is incumbent upon the Customer to know and follow procedures for the legal collection of sensitive information.

DATA PRIVACY

Qualtrics offers general-purpose research services where customers design surveys in an almost infinite number of ways. Since we only provide the platform for the software and Services—and don't delve into the Customer's specific implementation—we do not attest or know what Data are entered into our software. We realize that some companies wish to have more specific language regarding data privacy; however, our agreements and this white paper attest to our obligations to safeguard Data and to only process Data for performing the Services.

Being data agnostic is good for Customers, as Qualtrics protects all Data the same, without regard to its meaning or classification. Therefore, it's impossible for Qualtrics to perform in certain ways based on the classification declared by Customers. All Data are essentially invisible to us.

Certifications / Standards

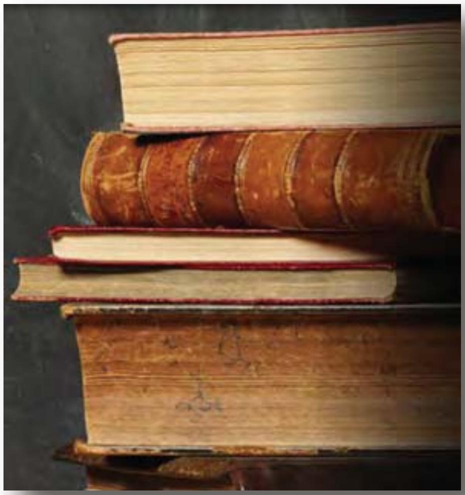

Qualtrics creates general purpose software products whereby the Customer owns and controls their Data and Users. Therefore, Qualtrics expressly disclaims any knowledge of the Data input to its Services, and cannot classify Data. All Data are considered highly confidential, treated equally, and protected using industry best security practices.

An analogy is when a person rents a storage unit. The storage company does not know what is placed in that space *(contents invisible)*. However, the company does have an obligation to provide adequate protection *(security controls)* so that no unauthorized person enters the premises *(data center)*. And the unit owner must secure the unit with a strong lock (*password and access controls)*.

That is why Qualtrics cannot sign any document that requires it to perform in certain ways based upon specific data types defined by a Customer or a government agency.

DATA CENTERS

All Qualtrics hardware (firewalls and servers) and data are located in tier 3 or tier 4 data centers, audited using the industry standard SSAE-16 Service Organization Control 1 (SOC-1) specification. Detailed reports may be requested by existing Customers either from the data center (listed above) or from Qualtrics with a signed confidentiality agreement. More details about data centers are discussed below.

ISO SECURITY STANDARDS

There are many security standards in which an organization may use to safeguard systems and data. Some, like IOS 2700x, were created by the International Organization for Standardization, and specify requirements and best practices for managing company and customer information. Qualtrics adheres to the principles set forth in these standards, including ISO 27001/2.

OPEN WEB APPLICATION SECURITY PROJECT (OWASP)

Qualtrics adheres to the OWASP ASVS methods for development and code review.

FEDRAMP: THE GOLD STANDARD

FedRAMP is considered the "gold standard" for security certifications.

In 2015, Qualtrics contracted with Veris Group to initiate the Federal Risk Authorization and Management Program (FedRAMP) accreditation, thus setting forth a roadmap to achieve one of the highest security ratings in the world.

The partnership with Veris Group provides expert guidance and an independent voice to assess the security posture of Qualtrics, ultimately benefiting its 9,000+ customers. Once Qualtrics is "FedRAMP Ready," another independent auditor will verify all the security controls written in the submitted documentation.

Presently, Qualtrics is FedRAMP "in Process." Please visit:

https://www.fedramp.gov/marketplace/in-process-systems/qualtrics-qualtrics-insight-platform/

The FedRAMP program was designed to enable U.S. federal agencies to utilize managed Internet-based service providers. The program is designed to comply with the Federal Information Security Management Act of 2002 (FISMA), and is based on the NIST 800-53 Rev. 4 standard with over 900 controls. Many controls may be cross-referenced with such standards as ISO 27001/2, PCI, and HIPAA (Hitech Act).

Security experts recognize that FedRAMP certification means having one of the highest non-military accreditations. It is supported by the Department of Defense, Department of Homeland Security, and GSA. Therefore, these methodologies and procedures will bring leading-edge security to all Qualtrics customers around the globe.

For more information, please visit the following link: http://www.fedramp.com

Human Resource Policies

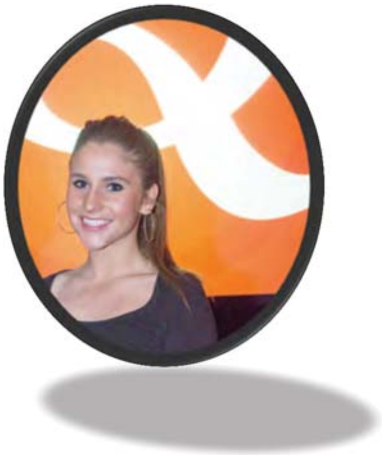

Qualtrics' rapid growth requires an influx of great talent. All new hires are held to rigorous standards and high qualifications. Qualtrics also requires background checks and adherence to strict privacy guidelines. Qualtrics is an equal opportunity employer.

CUSTOMER DATA ACCESS POLICY

Upon hire, all Qualtrics employees are required to sign a privacy and confidentiality agreement that specifically addresses the risks of dealing with sensitive information, including Customer accounts and Data. The policy includes the prohibition of access to Data without customer permission. This permission is typically granted for technical support only.

BACKGROUND CHECKS

Qualtrics performs background checks (to the extent permitted by local law) on applicants prior to employment.

PROVISIONING ACCESS

Access to Customer accounts is only given to those with a legitimate business need. This includes members of the Qualtrics support teams (QUni and Client Success), engineering team for specific debugging issues, and select members of our onboarding team that handle creating accounts for new customers. All system and service accesses are logged. **No employee has unfettered access to Customer Data.** More details below.

SECURITY THROUGHOUT QUALTRICS

Security isn't just a function of one team, but of the entire organization: engineering, IT, sales, support, HR, and legal departments. **Developers** are trained in coding best practices. The **InfoSec** engineers are responsible for securing and monitoring hardware at the data centers. This includes router/firewall configuration, cage security, and reliability verification. **TechOps** ensures desktop compliance and local server security. **Sales and support** may receive notice of a customer incident, and are responsible for escalating and investigating. **Onboarding/HR** staff are responsible for performing background employee checks, and ensuring new hires sign proper paperwork. The **Legal** team, comprising in-house and outside attorneys and security/privacy staff, ensures a safe work environment and maintains compliance with applicable laws.

Internal security audits are performed continuously via automated systems and verification that written policies are being followed. Qualtrics executives are notified of any abnormalities that would compromise company security or the safeguarding of Customer Data.

SECURITY AWARENESS TRAINING

Qualtrics employees are formally trained each year on company policies and security practices, and more frequently in email. This includes Security Awareness training and quarterly updates. All employees are instructed to immediately report possible security incidents to their manager, supervisor, and company director. The computer security section of the employee manual includes the following topics:

- Privacy law compliance
- Physical security
- Email acceptable use policy
- Access control
- Internet security
- Personal devices in the company
- Information Security Incidents
- Password policy and tips

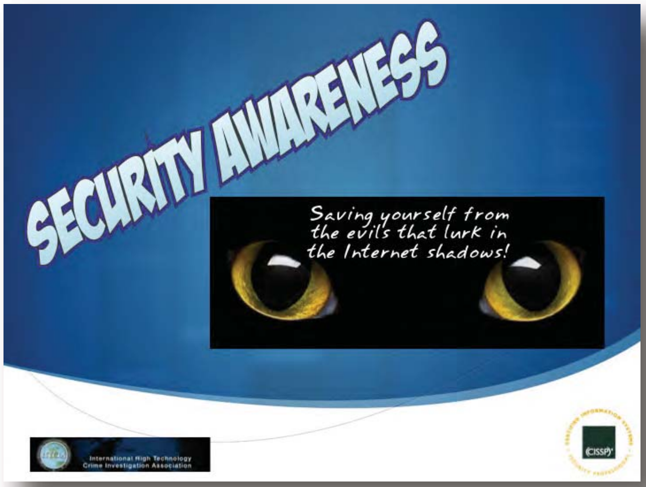

Network Design, Locations, and Assessments

DATA FLOW AND NETWORK DIAGRAM

In simple terms, transactions involve three parties—the Customer, the Respondents, and Qualtrics Services. The diagram below shows the interaction between these parties.

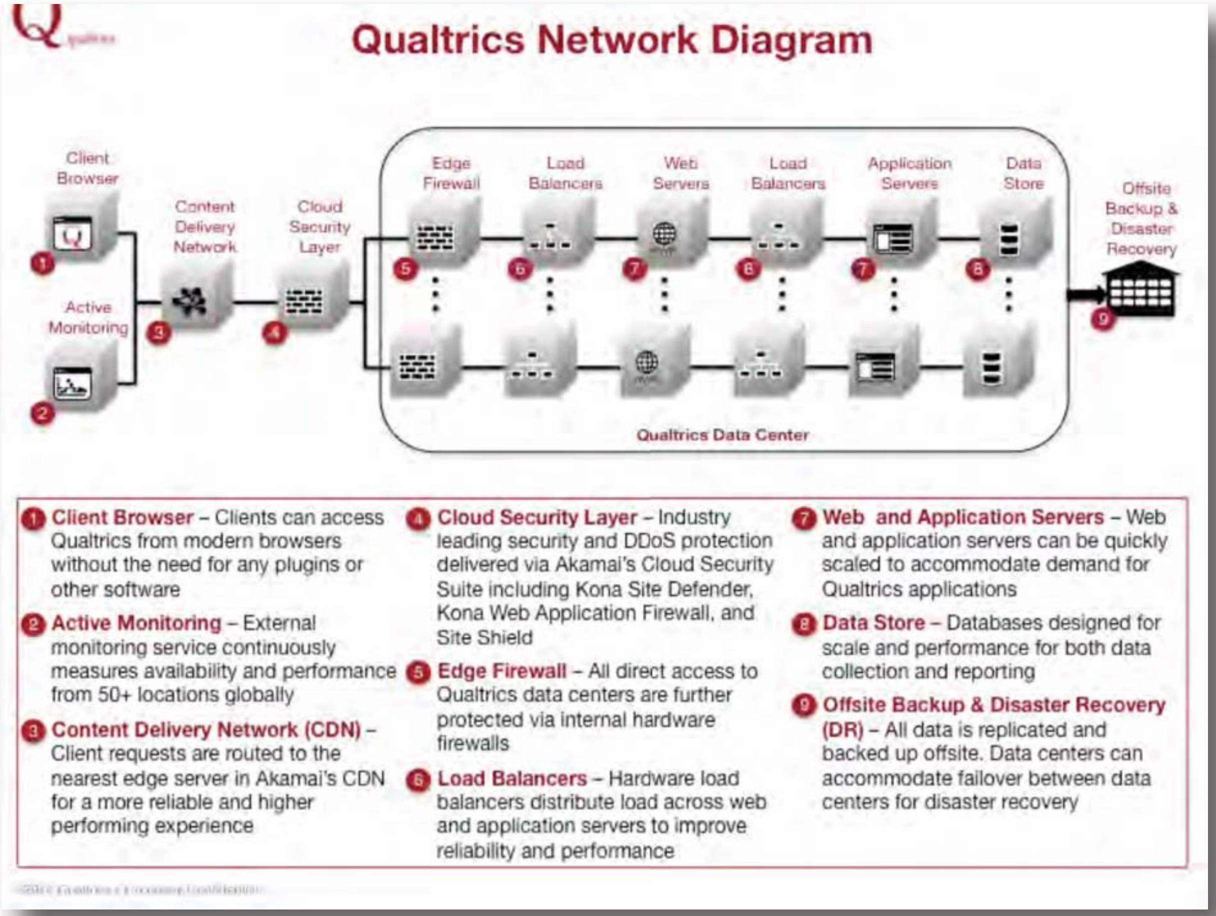

Respondents submit data using HTTPS (TLSv1.2 with AES 128/256 depending on the browser) to the front-end web server (typically *customername.*qualtrics.com). Data are processed by application servers and sent to database servers for storage. Web data are delivered to the Respondent in the form of survey questions, graphics, and other content created in the survey design. Some surveys are restricted by password or location, as setup by the survey creator. This multi-tiered architecture has multiple layers of hardware and software security to ensure that no device/user can be inserted into the communication channel.

For high availability and speed, base code and static images/docs are stored in the cloud and delivered to Users as efficiently as possible using cache and location information.

Users access the Qualtrics platform with login credentials using a web browser. Customers may choose to authenticate by linking their single sign-on (SSO) system to Qualtrics' Services. Brand Administrators have full

control over Users and the password policy.

QUALTRICS LOCATIONS

Qualtrics is a US-based company with secondary operations in Europe and Australia.

Qualtrics HQ— Headquarters is located in Provo, Utah against the backdrop of 13,000-foot Mount Timpanogos. All U.S. support staff are located here, as well as most salespersons and developers. The new facility officially opened in August 2016 by Governor Gary Herbert. All visitors to headquarters must show a government ID and be escorted. All employees are required to show ID badges upon entering the building.

The 200,000 square foot facility has full electronic access controls, CCTV, and monitored entry points. There is a backup generator in case of power failure. **No customer data are stored or processed at headquarters** or outside of security data centers, so even if the facility weren't operational, the software would still continue to function. And since Qualtrics uses ASP/SaaS software for most of its business operations, in the unlikely event of a major catastrophe, temporary offices could be setup in a few days; only power and Internet would be required. The Provo-Orem area is considered low risk for seismic activity, weather, and crime.

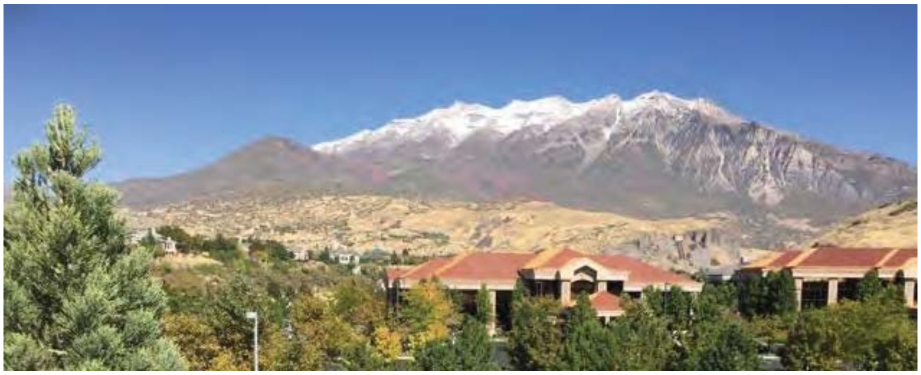

Qualtrics US (other locations)— The Seattle office is primarily for development. Dallas and Washington DC are satellite sales offices to accommodate local regions.

Qualtrics EU— Main office is located off Grafton street in the heart of Dublin, Republic of Ireland. It hosts multi-lingual sales and support staff. There are smaller sales offices in London and Munich.

Qualtrics AU— Located in Sydney, Australia. Supports Asia-Pac with sales persons and technical staff. There are small regional offices in Melbourne and Canberra.

DESKTOP POLICIES

Each component of our infrastructure (operating systems, desktops, routers, servers), both internal and in the data centers, have baselines that include security settings and default applications.

DATA CENTERS

Qualtrics leases space in two U.S. and one Canadian data centers. They are located in seismically low zones, and in areas least susceptible to mother nature's whims. Qualtrics owns and operates all server, firewall, and router hardware/software. Outside the U.S., Qualtrics uses co-location data centers with the hardware managed by the data center staff, but with the core operating systems and data always controlled by Qualtrics. *Data center personnel have no authorization to access Qualtrics data or underlying software environment* (as per contractual agreement and confirmed by SSAE-16 SOC audits).

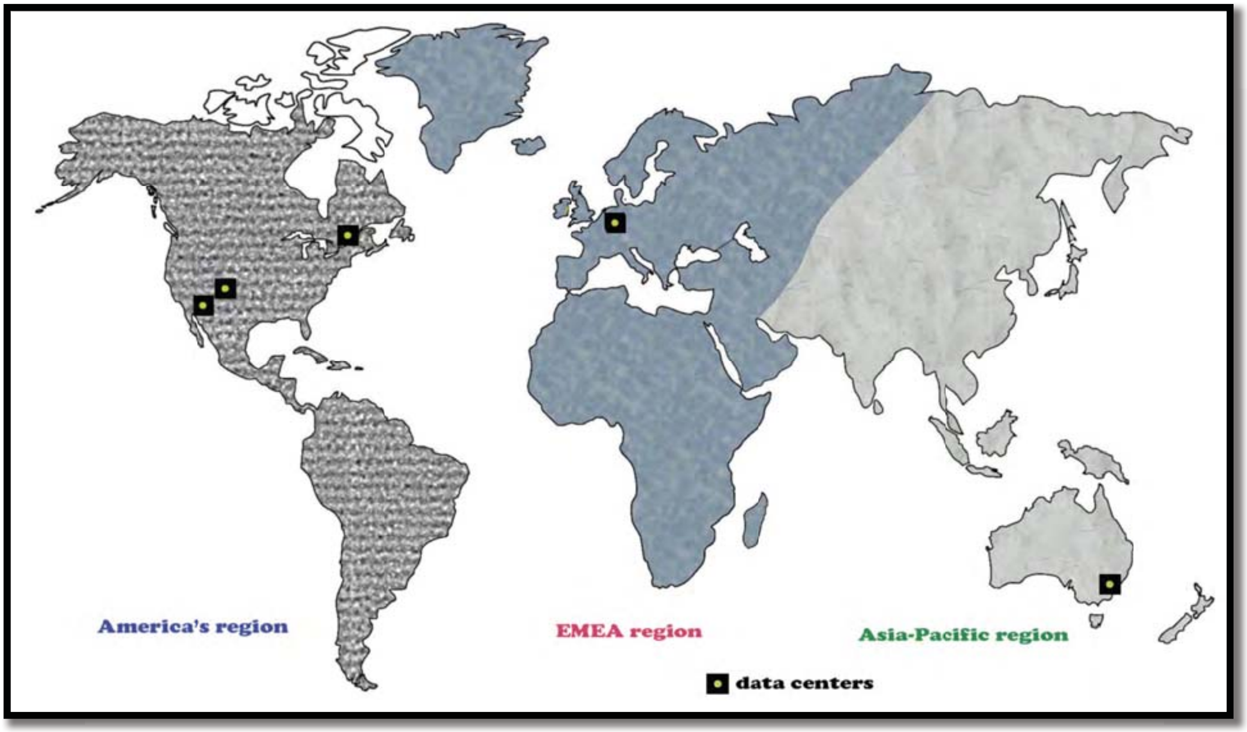

Equinix— A Tier-4 hosting facility located in Toronto, Canada.

Fortrust— A Tier-3 hosting facility located in Denver, Colorado.

IO Data Center— A Tier-3 hosting facility located in Phoenix, Arizona.

Amazon Web Service Data Centers— These highly secure facilities store Data for specific regions. Locations are Australia and the European Union. AWS provides the secure facility and hardware; it does not process information. All software is operated and controlled by Qualtrics.

Data-at-Rest Location—All Data are stored within the region where the Customer's primary data center resides, and will not be moved from that region. In other words, if a European customer has its data collected in the EU, its data will be stored and processed in EU. *Qualtrics does not transfer data unless requested by the Customer.*

Physical Access to Data Centers— Physical access to Data Centers is restricted to a limited number of employees, and includes the locked cage that houses the hardware used to provide the Services. Those employees do not have direct access to Customer Data.

Preventing Unauthorized Access

There is nothing more important to Qualtrics than protecting Customer Data. Qualtrics has implemented innovative methods to prevent unauthorized access to Data and the systems that host the Data. It starts with having documented security baselines for every component located in the data center, and ends with reinforcing security throughout the organization.

DATA STORAGE

Qualtrics Services use sophisticated databases that logically store Customer Data, as well as organize other components for quick retrieval and faster processing. To best optimize hardware and software, Customers share the same infrastructure, but Data are encoded so that only the correct Data will be sent to the requesting User. Access to Data requires direct ownership (the user who created the survey) or implied access (e.g. Brand Administrator or another User with access). Data at rest are encoded to prevent an unauthorized read access from deciphering the Data. For more advanced encryption, the Qualtrics Data Isolation feature uses AES-256 crypto, and is available for an additional fee.

SUPPORT ACCESS

Only qualified support staff has access to Customer accounts. This includes the QUni, Client Success, and engineering teams. The User must acknowledge the request for account access as shown in the screenshot below. All account access is logged and monitored by the InfoSec team. Each User account shows all login access.

PASSWORD POLICIES FOR THE SERVICES

This section applies to the Qualtrics Services, not the internal company network.

Qualtrics will never ask for any User password. All User passwords are hashed using BCrypt, and thus impossible to decode.

Failed Attempts— In order to block unauthorized access through password guessing, accounts are disabled after six invalid login attempts. Once an account has been deactivated, the account stays deactivated for ten minutes (and reset each time a new log in attempt is performed). The Brand Administrator may also re-activate the account.

Password Complexity— Qualtrics has a default five character minimum for user passwords. Settings for length, complexity, and periodic password expiration are available at the Brand level. For more complex passwords requirements, SSO integration is recommended.

Forgotten Password Policy— If a user forgets their password, or more than six invalid login attempts (causing their account to become deactivated), they may call Qualtrics support for help. There is also an *optional* self-service password reset option that sends an email with a link to create a new password.

Single Sign-On—SSO allows Customers to better control user management (additions/deletions) from the Customer's directory service, directly linked to the Qualtrics authentication service. Industry standard protocols are supported, including LDAP, SAML, OAuth, Token, and Shibboleth.

LOG FILES

Log files contain requestor IP address, protocol, request, result, and other info. Logs are stored on each device, and aggregated and analyzed using various tools. Monitoring systems trigger alarms and actions based on thresholds, thus helping to prevent an attack or other abuse.

Development Practices

The security of a platform hinges on developing solid and secure code. Weak code makes for a weak product. Here, we'll discuss our development practices.

DEVELOPMENT RELEASE CYCLE

Qualtrics uses an agile development model. This means that we take an iterative approach to software development and remain nimble in responding to the needs of our customers. Code is released on a two-week cycle that includes new features, bug fixes, and upgrades.

Each cycle includes comprehensive security checks to ensure that the code is vulnerability free. These checks include automated software assessments, peer and managerial reviews. The Software Development Life Cycle (SDLC) is shown below in the diagram. Sometimes this is referred to as "change and release control."

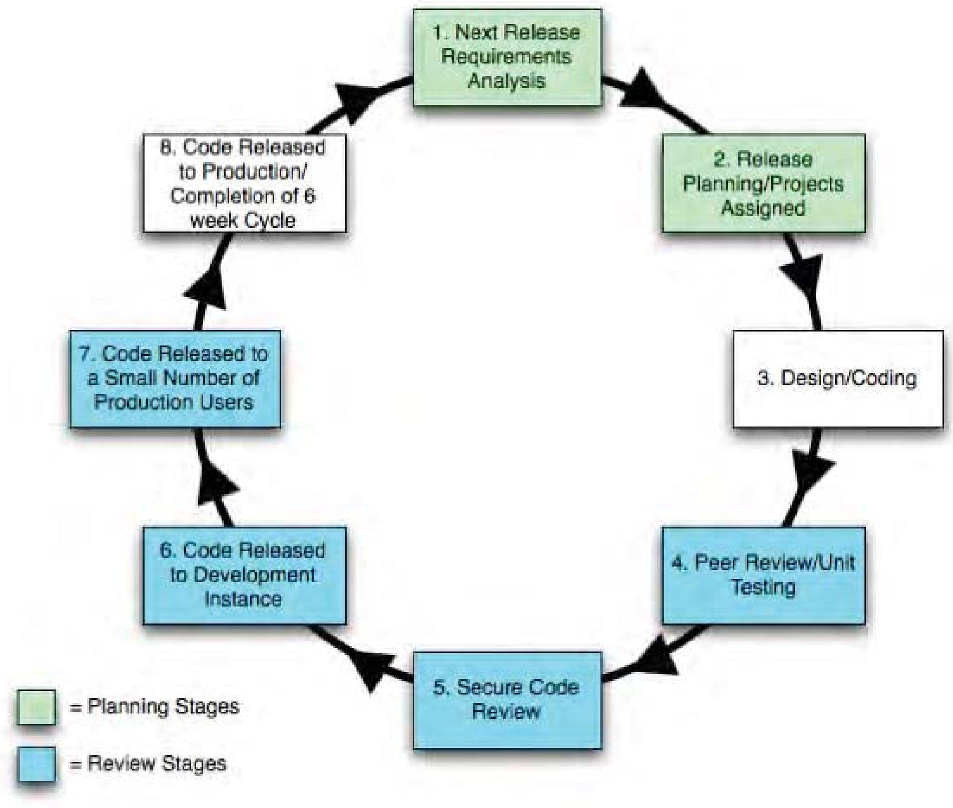

SECURE DEV WORKSTATIONS

Programmer workstations are Macintosh, and essentially terminals into the programming environment. Extra security is installed on these workstations, including a multi-factor login. This creates a solid operating environment where all code is stored and accessed securely, and access control is assigned to peers or managers for review.

SECURE CODE REVIEW

Programmers work individually or in pairs developing new code. As the end of each cycle approaches, code is peer-reviewed and tested in a staging environment completely separate from the production environment. This testing period eliminates most bugs before they could be introduced to production. No Customer Data are ever used in the testing environment. All code are scanned for vulnerabilities and syntax before deployment.

SEGREGATION OF RESPONSIBILITIES

There are many distinct Qualtrics programming teams, and each team is responsible for specific areas of the code. Engineers may only develop code in their area, and are not allowed to deploy that code to a production environment. This ensures a more secure and reliable development process.

STAGING (TESTING) ENVIRONMENT

All code is deployed and tested in a staging (test) environment that is functionally equivalent to the production environments. No Customer Data are used the staging environment.

DEPLOYMENT AND SECURITY PATCHES

Code is released outside of normal business hours (for a particular region), and with no downtime. If a security vulnerability is found, a fix may be deployed quickly without affecting the Service. Scheduled downtime is not required, although Qualtrics reserves the right to do so. Code is released by the deployment team to each data center on a schedule. All data centers are typically using the same release code within a few days.

Disaster Recovery

This section describes the Disaster Recovery Plan (DRP, that includes Data Loss Prevention or DLP) that the company will follow in the event of a disaster that would affect Data or the Services. A detailed internal document is used by engineers that contains specific details building, testing, and responding to disasters.

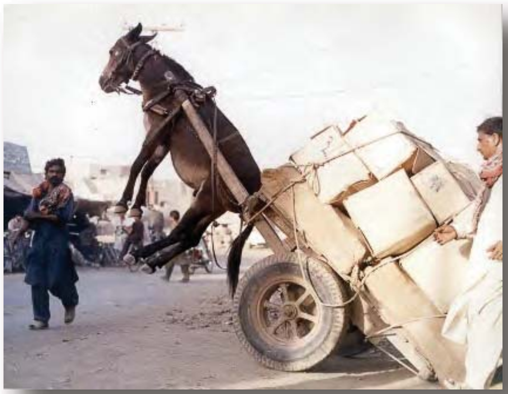

The purpose of the Disaster Recovery Plan is to ensure prompt and complete return to normalcy in the event of a disaster. The objectives of the plan are to ensure that, in event of disaster: 1) usability is restored promptly with little or no disruption to the User; and 2) Data loss is avoided due to extensive backup measures.

Disaster recovery and business continuity plans are tested bi-annually.

The Recovery Time Objective (RTO) is 24 hours to resume normal operations and Services. The Recovery Point Objective (RPO) is usually less than 4 hours to restore Customer Data. These times are estimates only.

POLICIES PERTAINING TO DRP

- Upon a disaster, Customer Data can be moved to a secure alternate location.
- Recovery processes are mainly automated, with no employee access to specific Customer Data.
- Data security and integrity must be monitored 24x7x365.
- Backup data must be kept in at least one secondary location.

IT DISASTER DECLARATION CRITERIA

In the event of an emergency, priorities include:

- preserving and recovering Data on database servers
- restoring functionality to infrastructure, including firewalls and web server
- restoring support and other operational servers

KEY DOCUMENTS AND PROCEDURES

There are various internal-only security policies that are specific to maintaining services and handling disasters and recovery.

Business Continuity

Qualtrics has an extensive Business Continuity plan in event of a disaster. Though details of the plan are internal only, below is a summary of how key business operations will operate following a disaster. This information supplements the information above in the Disaster Recovery section.

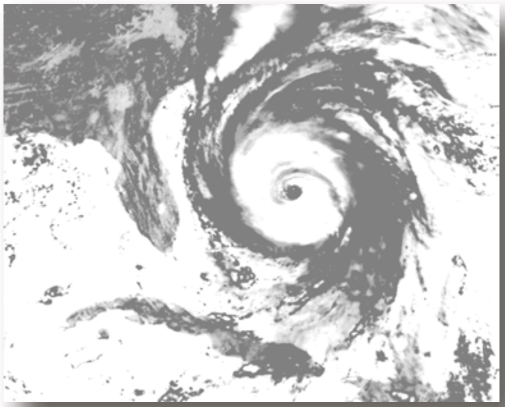

PURPOSE

The purpose of this business continuity plan is to ensure prompt and complete return to normalcy in the event of a service-affecting disaster.

GOALS AND OBJECTIVES

The objectives of this plan are to ensure that, in the event of a disaster all necessary support functions of the organization continue without undue delay. Data integrity and availability along with necessary support functions within the organization enable Qualtrics to maintain a trusting relationship with our Customers even in times of disasters.

REMEDIATION

Testing the BC plan is performed at least twice per year. Any significant findings are collected, and a report is produced for Engineering, TechOps, and InfoSec teams to review and create steps necessary to perform the test again and obtain a positive result. The VP of Engineering and Security Officer are also involved in the process.

POLICIES

All business continuity activities are coordinated with input from team leads and managers.

COMMUNICATION

Transparent communication, coupled with complete infrastructure/Systems redundancy, ensure successful continuity in times of disaster.

Backups – Data Retention

This section pertains to Data in the Services, not Qualtrics internal company retention procedures. All respondent Data are backed up by Qualtrics using two methods: automatic propagation across servers (immediate upon collection) and daily complete off-site encrypted backups. However, Customers are encouraged to back up their Data in case of accidental deletion/modification caused by one of their Users, and for their own archive/data retention policies.

AUTOMATIC PROPAGATION

Qualtrics uses advanced data storage technologies that record data to more than one physical device. This process is accomplished as soon as data is written, typically within a few seconds. It protects against storage device failure.

PERIODIC BACKUPS

Qualtrics performs a full daily backup of all production data. These backups are stored at alternate data centers in the same region where the data were created. Every backup file is encrypted using an advanced crypto method with a large key (for security reasons, details are not released).

DATA RETENTION

Since Customers/Users own and control their Data, they are responsible for accuracy, quality, integrity, legality, reliability, appropriateness, and intellectual property ownership of their Data. They are also responsible for backup (there are numerous download formats and mechanisms) and retaining the backup according to their retention policy. Depending on how active Data were deleted, it may be possible for the User to undelete it using a feature in the software. Once deleted Data are permanently inaccessible, then the User must restore from a personal backup. Survey definitions, response data, and some other data may be easily exported to the user's own system/device. *This is highly recommended as Qualtrics is under no obligation to restore Data not caused by its own negligence.*

Incident Response

An incident in this section refers to any discovery of a malfunction of the tool or a deliberate or accidental mishandling of Data (collectively, an "Incident"). Such Incidents require a quick response, and specific employees practice simulated CODE RED alerts. A detailed incident response policy is maintained by the InfoSec and Legal departments.

An Incident includes:

- A malfunction, disruption, or unlawful use of the Service;
- The loss or theft of Data from the Service;
- Attempts (either failed or successful) to gain unauthorized access to Data, information storage, or a computer system; or
- Material delays or the inability to use the Service
- Any event that triggers privacy notification rules, even if such an event is not due to Qualtrics' actions or inactions.

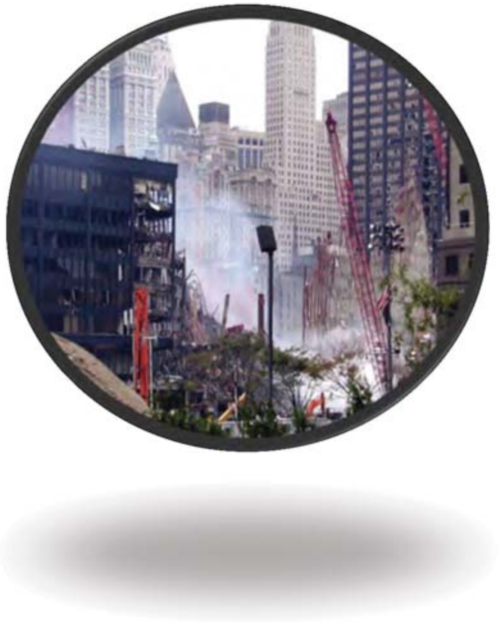

RESPONSE TEAM

The Qualtrics response team is comprised of members of its support and engineering teams who have expertise in technical issues, network security, and the software. The Engineer-on-call is available for emergency responses 24 hours a day 365 days a year.

NOTIFICATION REQUIREMENTS

An Incident involving personal data (as defined by applicable regulations or laws) may require certain notification procedures. Qualtrics has suitable policies to handle these requests, and has a team of outside attorneys, privacy staff, and security experts to respond to the particular notification needs based on the content disclosed.

Risk Management

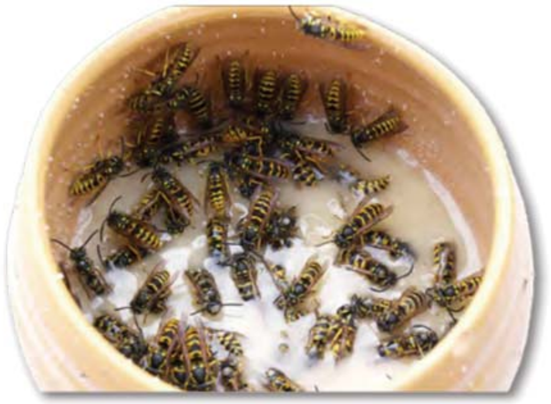

This section describes the risk management approach at Qualtrics: the underlying approach, the roles and responsibilities of the board, the senior management team, and other key parties. It also outlines key aspects of the risk management process, and identifies the main reporting procedures.

The following key principles outline Qualtrics' approach to risk management and policies.

1. The board and senior officers have responsibility for overseeing risk management within the company as a whole.
2. The senior management team supports, advises and implements policies approved by the board and officers.
3. The management recognizes and weighs the financial and non-financial implications of the risks.
4. Managers are responsible for encouraging good risk management practice within their department(s).
5. Key risk indicators will be identified and closely monitored on a regular basis.

RISK MANAGEMENT POLICIES

This broad set of policies encompasses a number of elements that together facilitate an effective and efficient operation, enabling Qualtrics to respond to a variety of operational, financial, and commercial risks. These elements include:

Policies and procedures

Monthly reporting

Business planning and budgeting

High level risk framework (significant risks only)

Employee risk frameworks

Internal audit program

External audit

Third party reports

OTHER SECURITY DOCUMENTS

Qualtrics customers with a "need to know" may request these documents provided there is valid reason and an executed confidentiality agreement in place as they contain detailed information about our operations.

Federal Standards White Paper, Security Assessment Questionnaire (SAQ), Standard Information Gathering (SIG) questionnaire, the Detailed Security Assessment, and CAIQ.

For more details regarding Qualtrics security, contact your account executive or Qualtrics support.

//AKM

Privacy Appendix

GENERAL INFORMATION

While Qualtrics agrees to follow applicable laws, most obligations in data privacy laws and regulations are for the data collector/controller. The collector/controller solely determines what data to collect, from whom, and from where. Qualtrics does not view, control or otherwise see the Data and therefore cannot classify, categorize or otherwise differentiate different types of Data. As a data processor, Qualtrics will process and safeguard all data equally without regard to what the data represents. Qualtrics cannot process any specific data differently.

Generally, Qualtrics meets or exceeds data processing requirements for privacy laws worldwide, and adheres to common principles. The full version of this Paper contains appendices specific to privacy laws in the US, EU, Australia, and Canada. **Qualtrics received self-certification in the Privacy Shield program by the U.S. Department of Commerce in December 2016.** A compliant privacy policy is posted at http://www.qualtrics.com/privacy. Qualtrics retains the American Arbitration Association/International Centre for Dispute Resolution should a dispute arise.

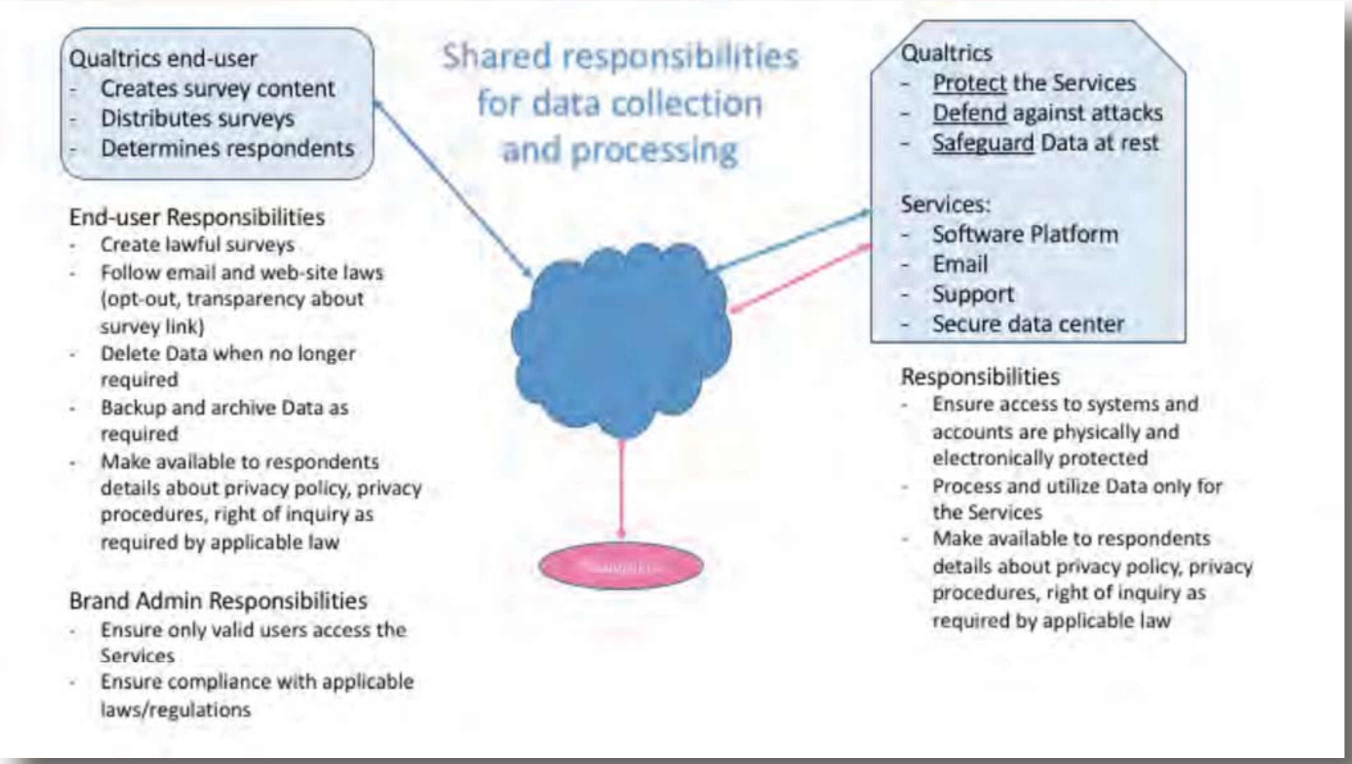

RESPONSIBILITIES

The diagram below briefly describes each party's responsibilities for collecting or processing Data.
