# Supplementary material for: Effectiveness of a Vaping Cessation Text Message Program Among Young Adult e-Cigarette Users: A Randomized Clinical Trial
Source: JAMA Intern Med. 2021 May 17;181(7):923–30. doi: 10.1001/jamainternmed.2021.1793 (PMC8129897; doi:10.1001/jamainternmed.2021.1793)
Supplement: Supplement 2. — eAppendix 1. Moderation Analyses eAppendix 2. Multiple Imputation Approach eTable 1. Comparison of Baseline Characteristics Between Non-Responders and Responders at 7-Month Follow-Up eTable 2. Sensitivity of Intervention Effects to Missing Data Assumptions eTable 3. Moderators of Intervention Effects on 30-day ITT Abstinence eReferences [file jamainternmed-e211793-s002.pdf]

Supplemental Online Content

Graham AL, Amato MS, Cha S, Jacobs MA, Bottcher MM, Papandonatos GD. Effectiveness of a vaping cessation text message program among young adult e-cigarette users: a randomized clinical trial. Published online May 17, 2021. *JAMA Intern Med*. doi:10.1001/jamainternmed.2021.1793

eAppendix 1. Moderation Analyses

eAppendix 2. Multiple Imputation Approach

eTable 1. Comparison of Baseline Characteristics Between Non-Responders and Responders at 7-Month Follow-Up

eTable 2. Sensitivity of Intervention Effects to Missing Data Assumptions

eTable 3. Moderators of Intervention Effects on 30-day ITT Abstinence

eReferences

This supplemental material has been provided by the authors to give readers additional information about their work.

© 2021 Graham AL et al. *JAMA Intern Med*.

eAppendix 1. Moderation Analyses

All baseline characteristics described in Table 1 were examined as potential moderators of the treatment effect on our primary outcome (30-day ITT abstinence rates at 7-month follow-up), one moderator at a time. Logistic regression models were fit that included main effects of treatment arm, baseline values of the moderator, and treatment by moderator interactions. Logistic regression coefficients (betas) corresponding to the interaction term, their standard errors and p-values are reported in eTable 2.

As the assessment-only control was used as the reference group in these analyses, betas represent treatment vs. control arm differences in the log-odds of abstinence per unit change in the moderator. This interpretation holds for both continuous and binary (0/1) moderators. For multi-category moderators, betas correspond to treatment vs. control arm differences in the log-odds of abstinence between the target and reference category of the moderator, e.g., in differences between Black vs. white racial categories.

© 2021 Graham AL et al. *JAMA Intern Med*.

eAppendix 2: Multiple Imputation Approach

We evaluated the sensitivity of our intent-to-treat (ITT) findings to the “missing = vaping” assumption using the multiple imputation (MI) approach of Hedeker et al.[1] which has been further evaluated and refined by others.[2-4] This approach is driven by the observation that in any 2x2 table that stratifies missing outcomes (yes/no) by vaping status (yes/no), the odds of vaping among non-respondents can be imputed by first estimating the odds of vaping among respondents and then multiplying them by the odds ratio capturing the association between missingness and vaping (OR.miss). The stronger the odds ratio, the more likely that the missing outcomes represent vaping. Conversely, the weaker the odds ratio, the more likely that the missing data represent abstinence. Two important cases occur when (a) OR.miss=1, i.e., there is no association between missingness and vaping outcome, and (b) OR.miss=+∞, in which case missing outcomes are certain to represent vaping. The first case corresponds to a Missing-at-Random (MAR) scenario, as defined by Little and Rubin[5] whereas the second case reduces to the ITT assumption. Of note, all OR.miss values other than 1 represent a non-ignorable missingness mechanism (NMAR), whose validity cannot actually be tested from the available data. For that reason, it is common to vary OR.miss over a large range (symmetric around 1 in the logarithmic scale) and to evaluate the sensitivity of the findings to the model assumptions.

To the extent that the odds of vaping among respondents are themselves a random variable subject to estimation uncertainty, multiple imputation is needed to fully propagate this uncertainty to the standard errors of the parameters of interest. In building an imputation model, it is worth noting that outcome missingness is allowed to depend on observed data under a MAR assumption. To make the MAR assumption more plausible, it is, therefore, typical to incorporate in the imputation model information from the baseline variables that have little missing data of their own. Following Hedeker[1] and Smolkowski[2], we chose to enrich our imputation model by estimating the odds of abstinence among respondents separately by nicotine dependence levels at baseline (vape within 30 minutes after waking vs. not). We also evaluated the strength of the (untestable) model assumptions by noting the extent to which it reduces the fraction of missing information (FMI) in the parameter of interest: the odds ratio capturing the association between the

© 2021 Graham AL et al. *JAMA Intern Med*.

text messaging intervention and vaping outcome (OR.vape). Under a complete case analyses (CCA), the observed missingness rate at 7-month follow-up was 24%, whereas ITT reduces this to zero by setting all missing outcomes to vaping (OR.miss=+∞). Therefore, we would expect finite values of OR.miss to reduce the FMI from 24% towards zero as they depart from the MAR value (OR.miss=1). The FMI is typically unknown, but can be estimated as the ratio of the between-imputation variance to the total variance of the parameter of interest, as the between imputation variance is zero in a scenario with no missing data.[6] To improve the accuracy of the FMI estimate, we used M=10,000 imputations for each value of OR.miss in our sensitivity analyses.

The MI findings are shown in eTable 2 as we varied OR.miss in a fashion symmetric around 1 in the logarithmic scale in a manner non-differential across study arms. As OR.miss increased from 1/100 to 100, the abstinence rates in the control (P0) and intervention (P1) arms both decreased towards their ITT estimates, with the difference shrinking monotonically from P1-P0 = 8.29% to 5.39%, indicating a smaller treatment effect as more non-respondents were classified as vapers. In contrast, using relative risk instead of risk difference as our preferred metric, increases in OR.miss led to an increase in the estimate of the treatment effect from P1/P0 = 1.20 to 1.29 at their ITT limit. Finally, use of an odds ratio scale showed intervention effects broadly symmetric around the MAR model, with OR.vape = [P1/(1-P1)]/(P0/(1-P0)] = 1.38-1.40 varying across a quite narrow range close to the ITT estimate of 1.39. Despite these differences, all 3 metrics resulted in positive and very highly significant estimates of the treatment effect. For robustness reasons, we chose to highlight the OR findings in the abstract. Regarding the FMI estimates, we note that they approach zero at both ends of the range (1/100, 100), indicating that more extreme values of OR.miss need not be explored. Indeed, the estimates at OR.miss=100 are indistinguishable from the ITT estimates. As for a Complete Case Analyses (CCA) that used treatment arm as the sole predictor of outcome, the estimated abstinence proportions appear to lie between imputation models that treat non-responders as 4/5-5/4 as likely to be vapers than abstainers.

© 2021 Graham AL et al. *JAMA Intern Med*.

eTable 1. Comparison of Baseline Characteristics Between Non-Responders and Responders at 7-Month Follow-Up					
	**Total**	**Non-Responder**	**Responder**	**P-Val**	**SMD**
	**N=2,588**	**N=621**	**N=1,967**		
Study condition=Control, n (%)	1284 (49.6)	290 (46.7)	994 (50.5)	.105	.077
Age, Mean (SD)	20.4 (1.7)	20.5 (1.8)	20.4 (1.7)	.012	.114
Gender, n (%)				.481	.056
Female	1303 (50.3)	300 (48.3)	1003 (51.0)		
Male	1253 (48.4)	314 (50.6)	939 (47.7)		
Non-binary or Other	26 (1.0)	6 (1.0)	20 (1.0)		
Race, n (%)				.001	.249
White	2159 (83.4)	547 (88.1)	1612 (82.0)		
Asian	123 (4.8)	15 (2.4)	108 (5.5)		
Black	38 (1.5)	2 (0.3)	36 (1.8)		
American Indian/Alaskan Native	18 (0.7)	5 (0.8)	13 (0.7)		
Multiracial	162 (6.3)	28 (4.5)	134 (6.8)		
Other	50 (1.9)	15 (2.4)	35 (1.8)		
Refused	38 (1.5)	9 (1.4)	29 (1.5)		
Ethnicity, Hispanic, n (%)	275 (10.6)	52 (8.4)	223 (11.3)	.047	.099
Sexual Minority, n (%)	493 (19.0)	100 (16.1)	393 (20.0)	.034	.103
Income, n (%)				.604	.063
Live comfortably	673 (26.0)	162 (26.1)	511 (26.0)		
Meet needs with a little left	1000 (38.6)	232 (37.4)	768 (39.0)		
Just meet basic expenses	778 (30.1)	198 (31.9)	580 (29.5)		
Don’t meet basic expenses	137 (5.3)	29 (4.7)	108 (5.5)		
Current student, n (%)	1932 (74.7)	409 (65.9)	1523 (77.4)	<.001	.259
Vaping frequency, nicotine, n (%)				.271	.077
Daily or almost daily	2410 (93.1)	587 (94.5)	1823 (92.7)		
Less than daily, but at least weekly	145 (5.6)	27 (4.3)	118 (6.0)		
Less than weekly, but at least monthly	33 (1.3)	7 (1.1)	26 (1.3)		
Time to first vape, within 30 minutes	2129 (82.3)	523 (84.2)	1606 (81.6)	.139	.058
Past year attempt to quit vaping, n (%)				.035	.134
None	222 (8.6)	64 (10.3)	158 (8.0)		
1-2 times	674 (26.0)	180 (29.0)	494 (25.1)		
3-5 times	911 (35.2)	198 (31.9)	713 (36.2)		
6 or more times	781 (30.2)	179 (28.8)	602 (30.6)		
Motivation to quit vaping, mean (SD)	4.54 (0.70)	4.55 (0.72)	4.53 (0.69)	.500	.031

© 2021 Graham AL et al. *JAMA Intern Med*.

Confidence to quit vaping, mean (SD)	3.47 (1.15)	3.43 (1.17)	3.48 (1.15)	.340	.044
No. closest friends that vape nicotine, M (SD)	2.91 (1.49)	3.00 (1.51)	2.89 (1.49)	.098	.076
Live with e-cig (nicotine) user, n (%)	1165 (45.0)	279 (44.9)	886 (45.0)	1.000	.001
Live with tobacco user, n (%)	916 (35.4)	223 (35.9)	693 (35.2)	.775	.015
Past 30-day use cigarettes, n (%)	841 (32.5)	200 (32.2)	641 (32.6)	.917	.007
Past 30-day use marijuana/cannabis, n (%)	1534 (59.3)	365 (57.8)	1169 (59.4)	.841	.011
Past 30-day binge drinking, n (%)	1929 (74.5)	464 (74.7)	1465 (74.5)	.784	.015
PHQ-2, score 3 or greater, n (%)	910 (35.2)	207 (33.3)	703 (35.7)	.307	.049
GAD-2, score 3 or greater, n (%)	1134 (43.8)	260 (41.9)	874 (44.4)	.295	.050

SMD: standardized mean difference
PHQ-2: Patient Health Questionnaire-2
GAD-2: Generalized Anxiety Disorder-2

© 2021 Graham AL et al. *JAMA Intern Med*.

eTable 2. Sensitivity of Intervention Effects to Missing Data Assumptions

OR.miss	FMI	P1	P0	Diff.vape	RR.vape	OR.vape	P-val
1/100	3	48.77	40.48	8.29	1.20	1.40	<.0001
1/20	9	46.69	38.65	8.04	1.21	1.39	.0001
1/10	14	43.85	36.31	7.54	1.21	1.37	.0002
1/5	19	40.48	33.33	7.14	1.21	1.36	.0006
1/3	21	37.44	30.56	6.88	1.23	1.36	.0011
1/2	21	35.23	28.57	6.66	1.23	1.36	.0014
2/3	21	33.48	27.01	6.47	1.24	1.36	.0016
4/5	20	32.25	25.93	6.32	1.24	1.36	.0017
1	19	30.98	24.81	6.16	1.25	1.36	.0018
5/4	18	30.32	24.24	6.08	1.25	1.36	.0018
3/2	17	28.98	23.08	5.90	1.26	1.36	.0018
2	15	28.28	22.48	5.80	1.26	1.36	.0017
3	12	27.00	21.26	5.74	1.27	1.37	.0015
5	9	25.51	20.00	5.51	1.28	1.37	.0012
10	5	24.88	19.36	5.53	1.29	1.38	.0010
20	3	24.88	19.36	5.53	1.29	1.38	.0009
100	1	24.09	18.70	5.39	1.29	1.38	.0008
ITT	0	24.08	18.63	5.45	1.29	1.39	.0007
CCA	24	32.27	24.04	8.23	1.34	1.51	.0001

OR.miss = assumed Odds Ratio capturing the association of vaping and survey non-response
FMI = Fraction of Missing Information in multiple imputation estimate of log(OR.vape)
P1 = abstinence rate (%) in intervention arm
P0 = abstinence rate (%) in assessment-only control arm
Diff.vape = P1-P0 = difference in abstinence rates between intervention and control arms
RR.vape = P1/P0 = abstinence Rate Ratio between intervention and control arms
OR.vape = [P1/(1-P1)]/(P0/(1-P0)] = Odds Ratio capturing the association of abstinence and intervention arm
ITT = Intention-to-Treat (OR.miss=+∞)
CCA= Complete Case Analysis

© 2021 Graham AL et al. *JAMA Intern Med*.

**eTable 3. Moderators of Intervention Effects on 30-day ITT Abstinence Rates.**			
	**Beta***	**Std. Error**	**P-value+**
Age	-.022	.057	.705
Gender (reference = Male)			*.321*
Female	.273	.195	.162
Non-binary or Other	-.366	.896	.682
Race (reference =White)			.116
Asian	-.241	.421	*.567*
Black	-.592	.674	*.379*
American Indian/Alaskan Native	-2.387	1.302	*.068*
Multiracial	.392	.383	*.306*
Other	-1.420	.767	*.064*
Refused	-.554	.946	*.558*
Ethnicity, Hispanic	-.386	.291	.186
Sexual Minority	.285	.250	.255
Income (reference = Live comfortably)			.951
Meet needs with a little left	.023	.244	*.926*
Just meet basic expenses	.025	.258	*.922*
Don't meet basic expenses	-.239	.467	*.609*
Current student	.305	.226	.178
Vaping frequency (reference = Daily/almost Daily)			.377
Less than daily, but at least weekly	-.143	.370	*.699*
Less than weekly, but at least monthly	-1.431	1.170	*.221*
Time to first vape, within 30 minutes	.267	.232	.250
Past year attempt to quit vaping (reference = none)			.290
1-2 times	.285	.398	*.474*
3-5 times	-.201	.380	*.597*
Motivation to quit vaping	.071	.152	.643
Confidence to quit vaping	-.138	.087	.113
No. closest friends that vape nicotine	.030	.064	.642
Live with e-cig (nicotine) user	-.183	.196	.348
Live with tobacco user	-.102	.201	.613
Past 30-day use cigarettes	-.021	.207	.919
Past 30-day use marijuana/cannabis	.088	.195	.653
Past 30-day binge drinking	.128	.229	.575
PHQ-2, score 3 or greater	-.002	.202	.993
GAD-2, score 3 or greater	.078	.195	.690
*TIQ vs. Control differences in abstinence rates measured in the log-odds ratio scale. Positive signs indicate that TIQ performed better than assessment-only control.			

© 2021 Graham AL et al. *JAMA Intern Med*.

+For multi-category factors, omnibus p-values test for TIQ moderation effects across all factor categories. They are supplemented by p-values in italics for category-specific comparisons vs. the reference group.

© 2021 Graham AL et al. *JAMA Intern Med*.

eREFERENCES

1. Hedeker D, Mermelstein RJ, Demirtas H. Analysis of binary outcomes with missing data: missing = smoking, last observation carried forward, and a little multiple imputation. *Addiction.* 2007;102(10):1564-1573. doi:10.1111/j.1360-0443.2007.01946.x.
2. Smolkowski K, Danaher BG, Seeley JR, Kosty DB, Severson HH. Modeling missing binary outcome data in a successful web-based smokeless tobacco cessation program. *Addiction.* 2010;105(6):1005-1015.
3. Jackson D, Mason D, White IR, Sutton S. An exploration of the missing data mechanism in an Internet based smoking cessation trial. *BMC Med Res Methodol.* 2012;12:157. doi:10.1186/1471-2288-12-157.
4. Jackson D, White IR, Mason D, Sutton S. A general method for handling missing binary outcome data in randomized controlled trials. *Addiction.* 2014;109(12):1986-1993. doi:10.1111/add.12721.
5. Little RA, Rubin DB. *Statistical Analysis with Missing Data.* New York, NY: Wiley; 1987.
6. Raghunathan T. *Missing Data Analysis in Practice.* Boca Raton, FL: CRC Press; 2016.

© 2021 Graham AL et al. *JAMA Intern Med*.
