# Supplementary material for: Effectiveness of a Vaping Cessation Text Message Program Among Young Adult e-Cigarette Users: A Randomized Clinical Trial
Source: JAMA Intern Med. 2021 May 17;181(7):923–30. doi: 10.1001/jamainternmed.2021.1793 (PMC8129897; doi:10.1001/jamainternmed.2021.1793)
Supplement: Supplement 3. — Data Sharing Statement. [file jamainternmed-e211793-s003.pdf]

Data Sharing Statement

Graham. Effectiveness of a Vaping Cessation Text Message Program Among Young Adult e-Cigarette Users. *JAMA Intern Med*. Published May 17, 2021. doi:10.1001/jamainternmed.2021.1793

Data
Data available: Yes
Data types: Deidentified participant data, Data dictionary
How to access data: agraham@truthinitiative.org
When available: With publication

Supporting Documents
Document types: Statistical/analytic code, Informed consent form
How to access documents: agraham@truthinitiative.org
When available: With publication

Additional Information
Who can access the data: Data will be made available to researchers whose proposed use of the data has been approved. Researchers must be primarily affiliated with a non-profit university or a non-profit research institution.
Types of analyses: Data will be made available for the sole purpose of replicating analyses reported in the manuscript.
Mechanisms of data availability: Requested data will be made available via a proposal that is approved by the investigator and a signing official of Truth Initiative that includes a signed data access agreement. Data made available will not include investigator support.
Any additional restrictions: None.
